# Short-term effects of continuous cover forestry on forest biomass production and biodiversity: Applying single-tree selection in forests dominated by *Picea abies*

**DOI:** 10.1007/s13280-022-01749-5

**Published:** 2022-06-04

**Authors:** Adam Ekholm, Petter Axelsson, Joakim Hjältén, Tomas Lundmark, Jörgen Sjögren

**Affiliations:** 1 Department of Wildlife, Fish, and Environmental Studies, Swedish Univ. of Agricultural Sciences, 901 83 Umeå, Sweden; 2 Department of Forest Ecology and Management, Swedish Univ. of Agricultural Sciences, 901 83 Umeå, Sweden

**Keywords:** Biodiversity, Boreal forest, *Picea abies*, Selection system, Single-tree selection, Uneven-aged forestry

## Abstract

The rotation forestry system provides high biomass production, but could also have a negative impact on species sensitive to disturbance. Continuous cover forestry (CCF) could contribute to solving these conflicting goals, but its feasibility in nutrient limited boreal forests is yet unresolved. In a unique experiment, we simultaneously assessed the short-term effect of single-tree selection on both biomass production and biodiversity (vascular plants, bryophytes, wood-inhabiting fungi), and tested fertilization as a way to mediate growth-biodiversity trade-offs. We found that unharvested stands and stands subjected to single-tree selection had a similar species assemblage of vascular plants, bryophytes, and wood-inhabiting fungi*.* Fertilization increased growth by 37% and induced shifts in two understory species (favoring the grass *Avenella flexuosa* and disfavoring the bryophyte *Hylocomium splendens*). We conclude that single-tree selection may become a useful tool to enhance biodiversity in managed forests.

**Supplementary Information:**

The online version contains supplementary material available at 10.1007/s13280-022-01749-5.

## Introduction

Global biodiversity is declining as evident from declining populations and high current extinctions rates (Pimm et al. 2014; Van Klink et al. 2020). Forests, which constitute 31% of the total land area (FAO 2020), are no exception and suffer from biodiversity loss in intensively managed areas (Paillet et al. 2010; Newbold et al. 2015). To enhance biodiversity and mitigate climate change, the European Union presented a new forest strategy with a focus on promoting alternative management methods to clear-cutting such as mixed species and continuous cover forestry (European Commission 2021).

The forest in Sweden has been actively used for a very long time. Until the mid-1950s, selective logging was common but abandoned in favor of the rotation forestry system, which proved superior in terms of growth and also facilitated forest planning with the aim of maintaining an even flow of timber. The rotation forestry system involves a repeated cycle of ground scarification, planting or natural regeneration using seed trees, one or several occasions of thinning and subsequent clear-cutting. This management regime has resulted in forests with a single-storied and even-aged structure (Kuuluvainen and Gauthier 2018), which is highly efficient at producing wood: Finland and Sweden together constitute 1.2% of the world’s total forest area but produced approximately 12.6% of the total pulp and 6.2% of the total sawn wood in the world during 2019 (FAO 2020, 2021). Yet, this has also led to a landscape-level shift from old to young forests (Kuuluvainen and Gauthier 2018) and loss of key elements important for biodiversity such as large diameter trees, dead wood, and multi-storied stands (Esseen et al. 1997; Östlund et al. 1997; Linder and Östlund 1998). Many red-listed species depend on these elements and suffer from a reduction of natural forests (Berg et al. 1994; Norden et al. 2013). In fact, about 1400 red-listed species in Sweden are negatively affected by clear-cutting (SLU Artdatabanken 2020).

Species inhabiting the boreal forest have for a long time adapted to a complex set of natural dynamics, which can be simplified into three categories based on disturbance intensity (Angelstam and Kuuluvainen 2004; Kuuluvainen and Aakala 2011): First, stand-replacing dynamics which is characterized by large-scale disturbances, such as intense forest fires and storms. Here, a high proportion of the trees die, which results in the regeneration of a new forest with an even-aged structure. Second, cohort dynamics which is driven by partial disturbance, such as low-intense fires and medium intense fires that cause a high mortality to specific age cohorts, resulting in a stand dominated by surviving cohorts. Third, gap and patch dynamics which is driven by local or small scale disturbances, such as self-thinning or local insect outbreaks. This results in an uneven-sized stand structure with a widespread diameter distribution, including trees in all sizes with a few large and many small trees (Kuuluvainen 1994).

A common view has been that stand-replacing disturbances have dominated in the boreal forests of Fennoscandia, which has motivated the use of the rotation forestry system (Fries et al. 1997; Mielikäinen and Hynynen 2003). However, this management is too simplistic and does not fully represent the complex disturbance-succession cycle of unmanaged forests (Kuuluvainen 2009). In addition, stand-replacing disturbances has been suggested to occur at a lower frequency than previously thought, whereas cohort and gap/patch dynamics are more common (Berglund and Kuuluvainen 2021). Thus, to promote biodiversity there is a need to explore alternative management methods that resemble a wider spectrum of the disturbance-succession cycle.

The selection system is a type of silviculture that falls under the definition of continuous cover forestry. Selection system is practiced in full-storied (i.e., inverse J-shape) forests dominated by *Picea abies* (L.) Karst. In this system, stands have no clear development phase (like regeneration or thinning) and maintains a similar structure over time. Logging, which is termed single-tree selection, occurs on a regular or a non-regular basis and removes only a subset of the standing volume, where logged trees are replaced by small naturally regenerated trees (for more details, see review by Lundqvist 2017).

Clear-cutting in the rotation forestry system is associated with a rapid turnover in species communities (Hylander and Weibull 2012; Rudolphi et al. 2014), whereas minor interventions similar to single-tree selection do not induce such turnover. For instance, Jalonen and Vanha-Majamaa (2001) found the short-term response of understory vegetation to change more after clear-cutting than single-tree selection. Similarly, Rudolphi et al. (2011) found a higher species richness of bryophytes on stumps in thinned than clear-cut stands. For beetles, Joelsson et al. (2017) found that single-tree selection maintained a similar composition as unharvested reference stands, whereas recently clear-cut stands had a different species composition. From a theoretical perspective, a more complex habitat can enhance biodiversity (e.g., Lassau and Hochuli 2004; St. Pierre and Kovalenko 2014), where high vertical heterogeneity (i.e., standard deviation of vegetation height) is especially important for forest biodiversity (Heidrich et al. 2020). Given the resemblance between selection system and gap dynamics, selection system has the potential to mitigate negative effects of silviculture on biodiversity.

In terms of yield, studies indicate a lower growth in selection system than in the rotation forestry system when applied in northern Europe (Tahvonen and Rämö 2016; Parkatti et al. 2019). Given that nitrogen is a limiting nutrient in many forest ecosystems (LeBauer and Treseder 2008), especially in northern Europe (Högberg et al. 2021), applying fertilizers could improve yield for two reasons. First, in the rotation forestry system, studies have demonstrated a higher yield after nitrogen addition in *P. abies* forests (e.g., Bergh et al. 1999; Jacobson and Pettersson 2001), except for very fertile stands (Bergh et al. 2014). The addition of nutrients expands the total leaf area (Axelsson and Axelsson 1986), increases photosynthetic rates (Roberntz and Stockfors 1998), and can also modify the carbon allocation from below-ground to the stemwood (Bergh et al. 1999). Secondly, apart from above-ground competition for light, below-ground competition for nutrients from larger trees can be a limiting factor for the establishment and growth of seedlings, for instance in *Pinus sylvestris* (Axelsson et al. 2014). Hence, potential loss in yield from applying selection system could be mitigated by the application of fertilizers, but this could also come with pronounced effects on biodiversity (Strengbom and Nordin 2008; Rodríguez et al. 2021).

In this study, we investigate the short-term response of single-tree selection with and without fertilization on biodiversity and yield. While previous experiments on continuous cover forestry have addressed growth and biodiversity in separate studies, we examine this within the same experimental setup. This is done by assessing production (tree growth, number of seedlings, and small trees) and biodiversity (wood-inhabiting fungi, vascular plants, and bryophytes) responses in plots that have been unlogged or subjected to single-tree selection with and without fertilization. Our experiment consists of eleven sites spanning a latitudinal gradient of 750 km in the boreal and hemiboreal region of Sweden. Thus, we intend to test the following hypotheses:

(i) Single-tree selection and fertilization will increase tree growth, resulting in the following order of tree growth among treatments: single-tree selection with fertilization > single-tree selection = unlogged, control stand.
(ii) Single-tree selection and fertilization will increase the number of seedlings and small trees.
(iii) Single-tree selection and fertilization will impact the vascular plant and bryophyte community, where species sensitive to nutrient availability and light conditions will change in abundance.
(iv) Given that wood-inhabiting fungi depend strongly on the availability of dead wood, we expected wood-inhabiting fungi to be governed by treatment effects on dead wood and not treatment per se. For example, via logging residues from harvest or from damages from machinery on dead wood on the forest floor.

## Materials and methods

### Experimental setup

**Fig. 1:**
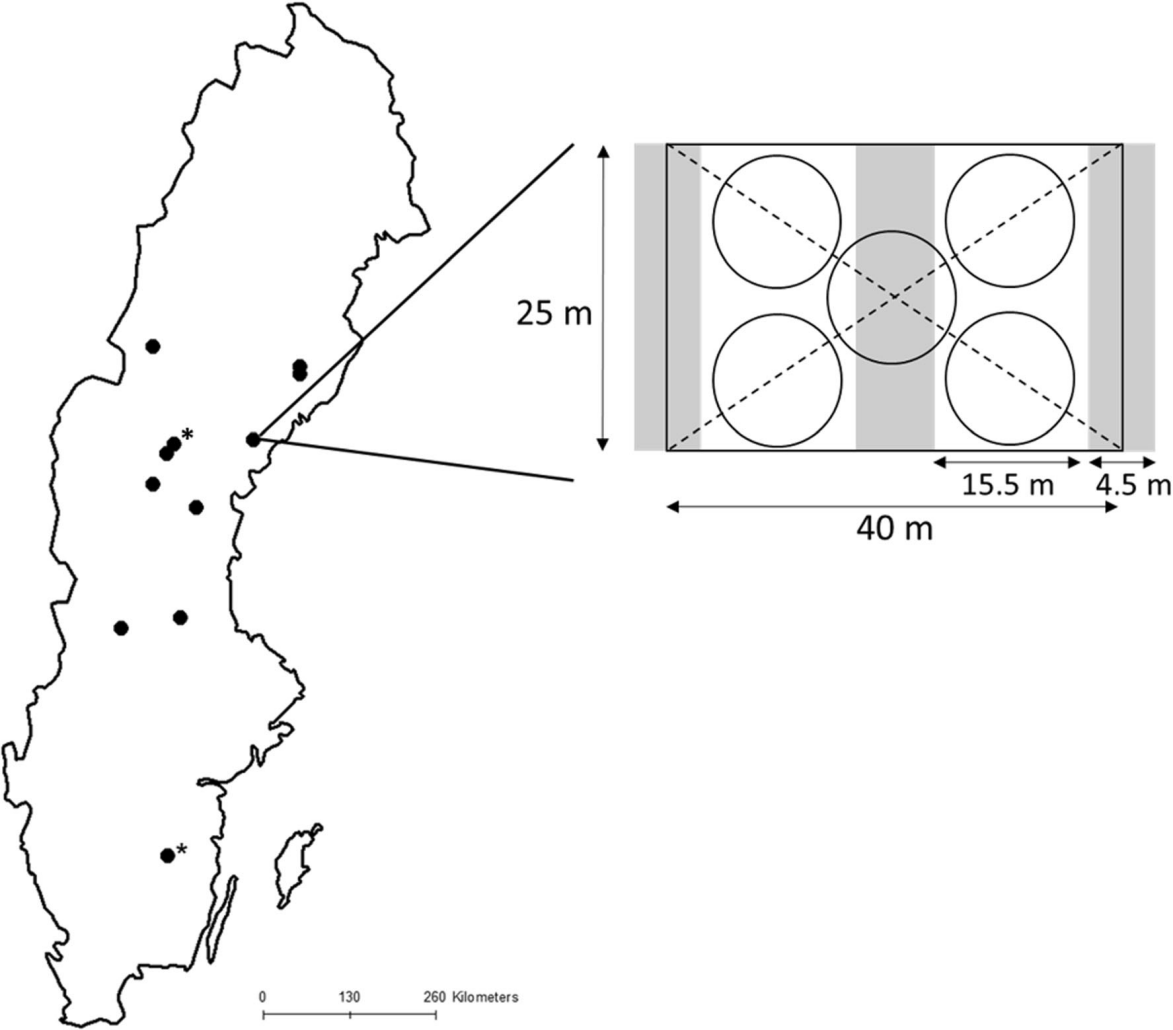
The spatial distribution of the eleven sites included in the study and the layout of the 25 × 40 m experimental plots (one site was 50 × 50 m). All sites have been surveyed for biodiversity and tree growth, except the two sites (marked with asterisks) which have only been surveyed for tree growth (Table S1). The gray area in the experimental plots represents strip cuttings and white areas parts that have been subjected to single-tree selection. Vegetation data were collected along the two diagonal lines (see methods for detailed description) and the number of *P. abies* seedlings (height 10–130 cm) was counted in each of the five 100 m^2^ circle

In this study, we established a before-after-control-impact experiment in eleven sites across a latitudinal gradient of 6° (750 km) in the hemiboreal/boreal region of Sweden (Fig. 1). Within each site, we established two or three adjacent 25 × 40 m plots with a 10 m unlogged buffer zone (the site of Kulbäcksliden had a plot size of 50 × 50 m). The forests were dominated by *Picea abies*, i.e., each plot contained at least 70% of *P. abies* (Fig. S1). Each plot was randomly subjected to single-tree selection with or without fertilization (Fig. 1; five sites lacked the fertilized treatment). A second or third plot was left unmanaged as a control plot. Single-tree selection was done in 2014 or 2015 and removed approximately 30% of the basal area (realized mean volume lost to logging and natural death was 34%, with a range of 23–64%). Since this was the first single-tree selection, most of the extracted volume came from creating strip roads and the rest from removing larger trees between strip roads. The diameter distribution was not full-storied (or following an inverse J-shape) in all plots before and after logging (Fig. S1A, B). Therefore, the operations in these stands are more similar to a thinning from above. But for simplicity, we only use the term single-tree selection in this paper. Fertilization was conducted in the first summer following single-tree selection in a subset (six out of the eleven) sites, using a single application of 150 kg N/Ha. This is standard practice in the rotation forestry and the application could be repeated by up to three times during a rotation (depending on the location of the stand; Högberg et al. 2014). The fertilization was carried out manually to ensure an even distribution of the fertilizer. Two sites had an additional replicate of the single-tree selection treatment without fertilization and one of these sites was particularly affected by a storm during the experimental period, which resulted in a higher removal of wood biomass in one plot (64% of the volume). Within the same site, bark was removed from storm-felled trees to reduce the risk of bark beetle infestation. For more details on the type of surveys done at each location, see Table S1.

### Production

#### Tree growth

To study how single-tree selection and fertilization influenced tree growth, we followed tree growth in eleven sites by using the method described in Karlsson (2003). In short, this meant that all trees with a diameter at breast height (dbh, 1.3 m above the germination point) > 5 cm were marked and cross-calipered both before logging in 2013–2014 (logging was conducted in either 2014 or 2015, which was 1–2 years after the first measurement) and again after logging in 2019. Smaller trees (dbh 0.5–4.9 cm) were also measured but not individually marked. Since the time of the first dbh measurement spanned between May and October, we set 1st of July as a limit, if measurements were done prior to this date we assumed a growing season of *n* + 1 years, otherwise a growing season of *n* years. This resulted in five to seven growing seasons between measurements. To calculate both the individual tree and stand volume in each plot, we measured the dbh, height to the living crown, and tree height on a set of sample trees (bark width was measured on some tree species). As measurements on bark width and height to the living crown were missing on a few sample trees, we estimated this with a linear regression using all sample trees on each site. For the most common tree species (*P. abies, P. sylvestris, Betula *sp.), we used the volume functions described in Brandel (1990) to calculate the volume of each sample tree. For other less common tree species (e.g., *Alnus glutinosa, Salix caprea*), we used functions described in Eriksson (1973) and Hagberg and Matérn (1975) (all volume functions are summarized in Karlsson et al. (2012)). These data were then used to establish a local relationship, in diameter classes of 2 cm, where the volume of a cross-calipered tree *V*_*t*_ is determined by $V_{t} = V_{s} * \frac{{d^{2} }}{{d_{s}^{2} }}$, where *V*_*s*_ is the mean volume of the sample trees, *d*^2^ is the squared diameter of the cross-calipered tree, and $d^{2} _{{\text{s}}}$ is the squared mean diameter of the sample trees (Karlsson et al. 2012).

#### Seedlings and small trees

We assessed the number of seedlings by surveying all trees (*Picea abies*, *Betula *sp.,* Sorbus aucuparia* L.,* Pinus sylvestris* L.,* Salix *sp.*/Populus tremula* L.) with a height ranging between 10 and 130 cm in five circular 100 m^2^ subplots within each treatment (Fig. 1). Then to study small trees (*n* = 11 sites) of *P. abies*, we counted all *P. abies* in each main plot with a dbh between 0.5 and 4.9 cm.

### Biodiversity

To assess the response of biodiversity to single-tree selection with and without fertilization, we did three surveys: (i) a pinpoint survey to estimate the abundance of bryophytes and vascular plants, (ii) an extensive presence/absence survey of bryophyte species in each plot, and (iii) a survey of all fruiting bodies of wood-inhabiting fungi in each plot. Each survey followed a before-after-control-impact design (BACI; Smith 2002) in a subset of sites (*n* = 9 sites; Table S1 before logging (2013–2014) and again in 2018 after logging (for species list, see Appendix S2).

By sampling control and treatment sites prior and after an environmental disturbance, BACI has the advantage of determining treatment effects relative to undisturbed control sites (detected by a significant interaction between treatment and time). The most intuitive example of an effect is when the control and treatment are similar before the disturbance and only the treated site changes after the disturbance (see e.g., control vs. fertilized treatment in Fig. 5d). Although it is desirable that pre-treatment levels are similar in both control and treatments, this is not a strict requirement in BACI studies making this methodology particularly appropriate for ecological studies in the field (see e.g., Chevalier et al. 2019). Furthermore, interactions can be manifested in various, complicated forms. For instance, a reduction in the control but not in the treatments could be difficult to understand. Such a pattern could for instance be interpreted as long-term negative trends being mitigated by the treatment (Chevalier et al. 2019).

In comparison to other experimental designs, where the treated plots are surveyed before and after (BA) or when a treated plot is compared to a control after treatment (CI), BACI is much more accurate at estimating true effects and the magnitude of these effects. On the other hand, BACI studies are more sampling intensive and the advantage of detecting significant effects is reduced at low replications (De Palma et al. 2018; Christie et al. 2019).

#### Abundance of vascular plants and bryophytes

To get an abundance estimate of species in the bottom layer (bryophytes) and field layer (vascular plants), we established two diagonal transect in each plot (Fig. 1). Each diagonal consisted of 200 randomly placed points that was spaced with at least a decimeter, resulting in 400 points per plot (realized value was 386–400 points). At each point, we penetrated the bottom layer and field layer with a stick (∅ = 5 mm) and noted the first species touching the stick in each layer. We also noted when there was no contact in the field layer and when the contact in the bottom layer was with bare ground, dead wood, water, or harvest residues. At the site of Kulbäcksliden, the before survey was done at 50 × 50 m plots and after at 25 × 40 m plots. Since we are interested in the relative abundance change, i.e., the interaction between time and treatment, effects of treatment can still be detected in spite of the area difference.

#### Bryophyte richness and functional groups

To further study if the assemblage, species richness and functional groups of bryophytes were influenced by single-tree selection and fertilization, we also identified all bryophyte species present in each plot (i.e., a presence/absence survey). Bryophytes that were not possible to determine in the field were collected and determined in the lab. One person spent in median 90 min per plot, with a total range of 60–145 min. To assign species to different functional groups, we used the bryophyte trait database described in Bernhardt‐Römermann et al. (2018). We divided bryophyte species into four functional groups according to two traits: habitat preference (M1.1 = “Largely restricted to closed forests,” M2.1 = “Occurs in forests as well as open land”) and light sensitivity (3–5 = Shade—semi-shade places, 7–9 = partly shaded—well-lit places). Since liverworts can be particularly sensitive to logging (Fenton et al. 2003; Dynesius 2015), we also included this group in the analysis. At the site of Kulbäcksliden, this survey and the survey described below were conducted at 25 × 40 m plots.

#### Wood-inhabiting fungi

To study wood-inhabiting fungi, we surveyed all fruiting bodies (both alive and dead) that could easily be identified in the field (some were determined in the lab). We did this by measuring all DW objects with a base diameter of ≥ 10 cm and a length of ≥ 150 cm (base had to be in the plot) in each plot and by counting the number of wood-inhabiting fungi species inhabiting each DW object. Since different species of wood-inhabiting fungi prefer dead wood with different decay stages, we noted the decay stage according to the Swedish national forest inventory (1 = Hard, 2 = Partly decayed, 3 = decayed, 4 = Much decayed; (Anonymous 2021)) and also the tree species and type of dead wood (standing or lying).

### Statistical methods

All analyses were done in the statistical program R ver. 4.0.1 (R Core Team 2021). As our model framework, we used generalized or linear mixed models with the function *glmer*, *lmer*, or *lme* from package *lme4* and *nlme* (Bates et al. 2014; Pinheiro et al. 2021). In the analyses described below, we model all response variables (except tree growth) as a function of treatment, time (before and after logging), and the interaction. To account for differences among sites, between plots and for repeated measures of the same plot, we included site as a random effect and nested plot under site. If the model displayed singularity, we removed site as a random effect.

For interactions with a *p* value ≤ 0.1, we used a post hoc test with Bonferroni corrections of *p* values for multiple comparisons to resolve differences within each level of the fixed effects (difference among treatments in the tree growth analysis and difference between before and after treatment in the biodiversity analysis).

Unless otherwise is stated below, we used a normal distribution in all models. All model residuals were visually inspected for normality and tested with Levene’s test for homogeneity of variance. If heterogeneity was detected, we applied a separate variance function using *varIdent* in combination with the function *lme* for linear mixed models (Pinheiro et al. 2021). Significance was tested with a marginal or type 3 ANOVA.

#### Hypothesis 1: Tree growth

To determine if single-tree selection and fertilization influenced tree growth on the plot level, we included all tree species and calculated (i) the relative volume growth rate, and (ii) the absolute volume and basal area growth (the latter log-transformed). The relative growth rate was calculated as ${\text{Plot}} = \sqrt[n]{{\frac{Total\, volume\, after}{{Total\, volume\, before}}}}$, where volume refers to the plot-level volume at the first survey (before: including all living trees) and second survey (after: including all living trees and trees that had died since the first survey), while *n* is the number of growing seasons between measurements. The absolute plot-level growth was obtained by dividing the total volume and basal area gain with the number of growing seasons. Growth was modeled as a function of treatment with site as a random effect. Standing volume or basal area after logging (summed over all trees with a dbh > 4.9 cm) was added as a covariate for absolute growth since it can influence growth (Lundqvist 2017).

Then, to determine how trees of different sizes (measured as volume and dbh) responded to treatment, we calculated the relative tree-specific annual growth rate of *P. abies*: ${\text{Tree}} = \sqrt[n]{{\frac{Volume\, or\, dbh\, after}{{Volume\, or\, dbh\, before}}}}$, where volume and dbh refer to the tree-specific volume or mean dbh at the first (before) and second (after) survey, while *n* is the number of growing seasons between measurements. We then modeled tree-specific annual growth rate as a function of treatment, dbh class (50–100, 100–150, 150–200, 200–250, > 250 mm) and the interaction (one tree was cross-calipered with a dbh of 16 mm and 157 mm, which was deemed unrealistic and subsequently removed from the tree-specific analysis).

#### Hypothesis 2: Seedlings and small trees

To examine the number of seedlings and small trees of *P. abies*, we modeled the response of *P. abies* in two size classes (seedlings: height 10–130 cm, small trees: dbh 0.5–0.49 cm, see above) and the Shannon diversity index of seedlings. Seedlings from each subplot were pooled at the plot level. For small trees we controlled for variation in plot size, that is, in the larger 50 × 50 m plot, we divided the number of small trees with 2.5. At the two sites which had an extra plot of the single-tree selection, we used the average number of small trees from both plots in the analysis.

#### Hypothesis 3: Vascular plants and bryophytes

To examine vascular plants, we used abundance data obtained from the survey of the diagonal transects (“Abundance of vascular plants and bryophytes” section) and modeled species richness and abundance of vascular plants with an average occurrence > 5% across the whole data set. To examine bryophytes, we used the survey of abundance (“Abundance of vascular plants and bryophytes” section) to model species abundances (> 5% across the whole data set) and the survey of presence/absence (“Bryophyte richness and functional groups” section) to model species richness and number of species in each functional group (according to habitat preference and light sensitivity) and number of liverwort species (Bernhardt‐Römermann et al. 2018). For bryophyte species richness, we removed uncertain observations and only included species that were possible to determine to the species level (except for two genera where no further determination was done: *Distichium* spp., *Brachythecium* spp., and complexes such as, e.g., *Dicranum fuscescens s.lat*, each genera/complex was counted as one species). We only included species that were described in the bryophyte trait database when analyzing the number of species within each functional group. Species richness and number of species in functional groups were analyzed as count data with a log + 1-transformation, whereas the abundance data were analyzed as percent. Using linear models for fitting count data is useful for complex models, i.e., when implementing random effects (Warton et al. 2016).

To assess the response of both the bryophyte and vascular plant assemblage, we conducted two multivariate PERMANOVA analyses (excluding species only present at a single plot) with 999 permutations. Here, we modeled the vascular plant assemblage from the abundance survey (“Abundance of vascular plants and bryophytes” section; data were fourth-root transformed) and the bryophyte assemblage using the survey of species presence/absence (“Bryophyte richness and functional groups” section; also excluding species present on all plots). Each response was modeled as a function of treatment and time, with the function *adonis2* from the Vegan package (Oksanen et al. 2020). We used the Bray–Curtis dissimilarity index to calculate matrix distances and the function *betadisper* to assess the homogeneity of variance (dispersion) among the predictor variables. To visually display potential differences in community composition, we created a two-dimensional NMDS (non-metric multidimensional scaling) plot before and after logging, again using the Bray–Curtis dissimilarity index with the function *metaMDS* from the vegan package (Oksanen et al. 2020).

#### Hypothesis 4: Wood-inhabiting fungi

To examine the response of wood-inhabiting fungi to treatment, we first studied the response of DW. Since 84% of the DW volume consisted of *P. abies* and *Pinus sylvestris*, we excluded all other species and modeled DW richness (log + 1-transformed) and volume (log-transformed) as a function of treatment and time. Dead wood richness was assessed by counting the number of different combinations of diameter (10–20, 20–30, > 30 cm), species (*P. abies, P. sylvestris*), type (standing or lying), and decay class in each plot. Volume was calculated for each DW, assuming the shape of a truncated cone: $= \frac{{l * \pi *\left( {r_{{{\text{max}}}}^{2} + r_{{{\text{max}}}} * r_{{{\text{min}}}} + r_{{{\text{min}}}}^{2} } \right) }}{3}$, where *V* is the volume of the object, *l* is the length, *r*_max_ is the radius at the base, and *r*_min_ is the radius at the top. At one plot, we lacked length information for five storm-felled trees that had been cut to smaller logs. For these DW, we used the same volume as DW of similar size within the same stand (a maximum deviation of 2 cm in base diameter and 2 cm in the top radius was allowed). To assess the response of wood-inhabiting fungi to logging treatment, we modeled species richness (log + 1-transformed) and DW occupancy (presence/absence of wood-inhabiting fungi on a dead wood unit) as a function of treatment and time. For species richness, we added the plot-level DW volume squared as a covariate, whereas the squared DW volume and decay class was added when analyzing occupancy. Occupancy was modeled as a logistic regression with a binomial distribution.

Data has been uploaded at the dryad digital repository https://urldefense.com/v3/_https://doi.org/10.5061/dryad.9cnp5hqmp_;!!NLFGqXoFfo8MMQ!pdoQOLNyP9Omcq78qYfJoFxZG0BjvsozQ8HgjSK28yHM69br6aGQIeVQPufzMrf_igCVN6VDaIAznDnyfdX-oPQmG2jF$

## Results

### Production

#### Hypothesis 1: Tree growth

**Fig. 2:**
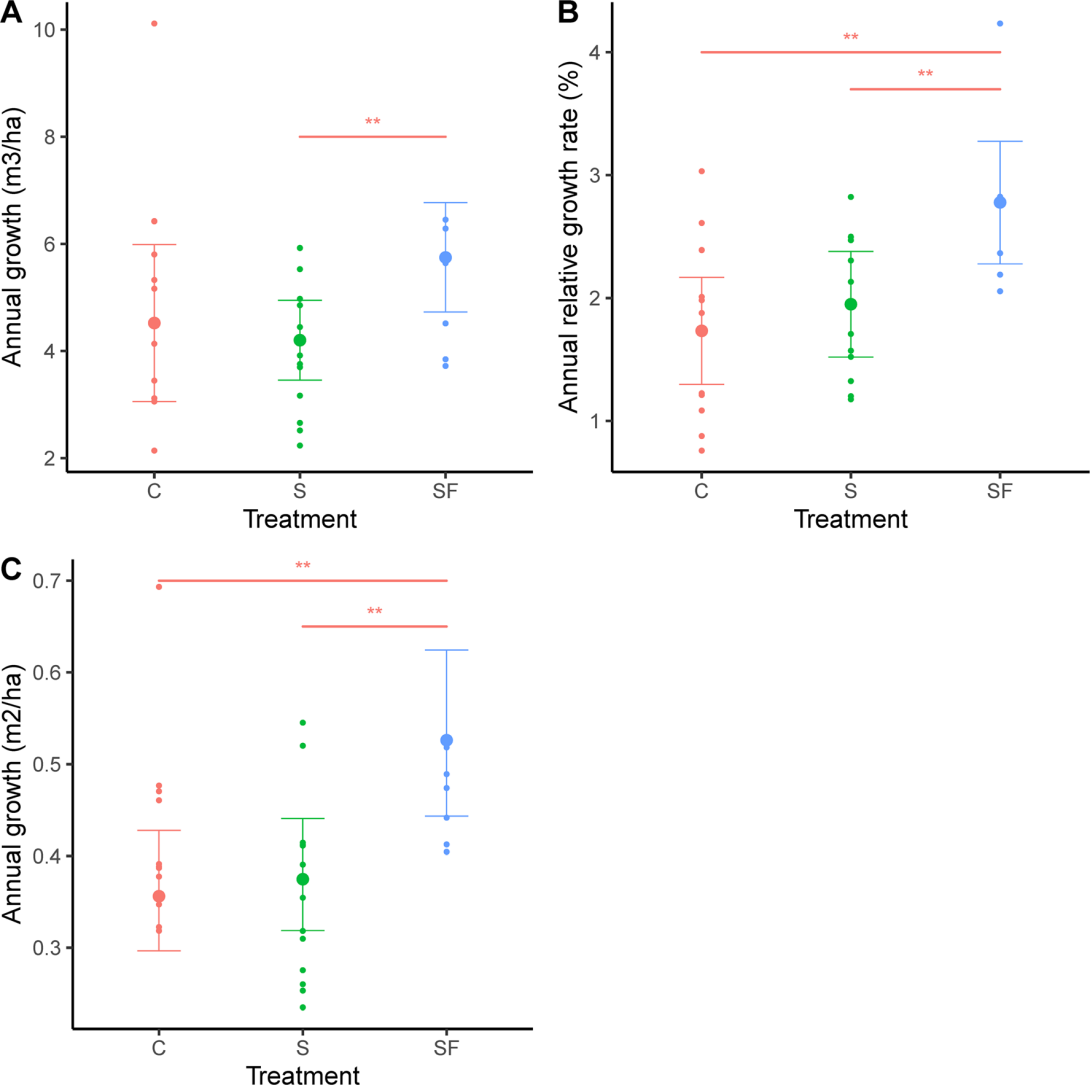
The absolute (**A**, **C**) and relative (**B**) annual plot-level volume (**A**, **B**) and basal area (**C**) growth in all three treatments (*C* control, *S* single-tree selection, *SF* single-tree selection with fertilization). Shown are least square means with 95% confidence intervals. Pairwise significant differences between treatments are denoted with an asterisk on a horizontal line (*p = 0.05–0.10, *p = 0.01–0.05, **p < 0.01). Basal area growth is back-transformed from a log-transformation. Raw data displayed in the background

At the plot level, the annual growth differed among treatments (Volume: *F*_2, 16_ = 8.08, *p* < 0.01; Basal area: *F*_2, 18.21_ = 20.31, *p* < 0.01; Relative volume: *F*_2, 17.55_ = 13.16, *p* < 0.01; Fig. 2). The post hoc Bonferroni test revealed that the plot-level volume and basal area growth was consistently higher in the fertilized treatment (Fig. 2; Table S2). Furthermore, absolute volume growth increased with standing volume (*F*_1, 16_ = 6.41 *p* = 0.02) and basal area growth increased with basal area (*F*_1, 20.91_ = 6.99, *p* = 0.02).

**Fig. 3:**
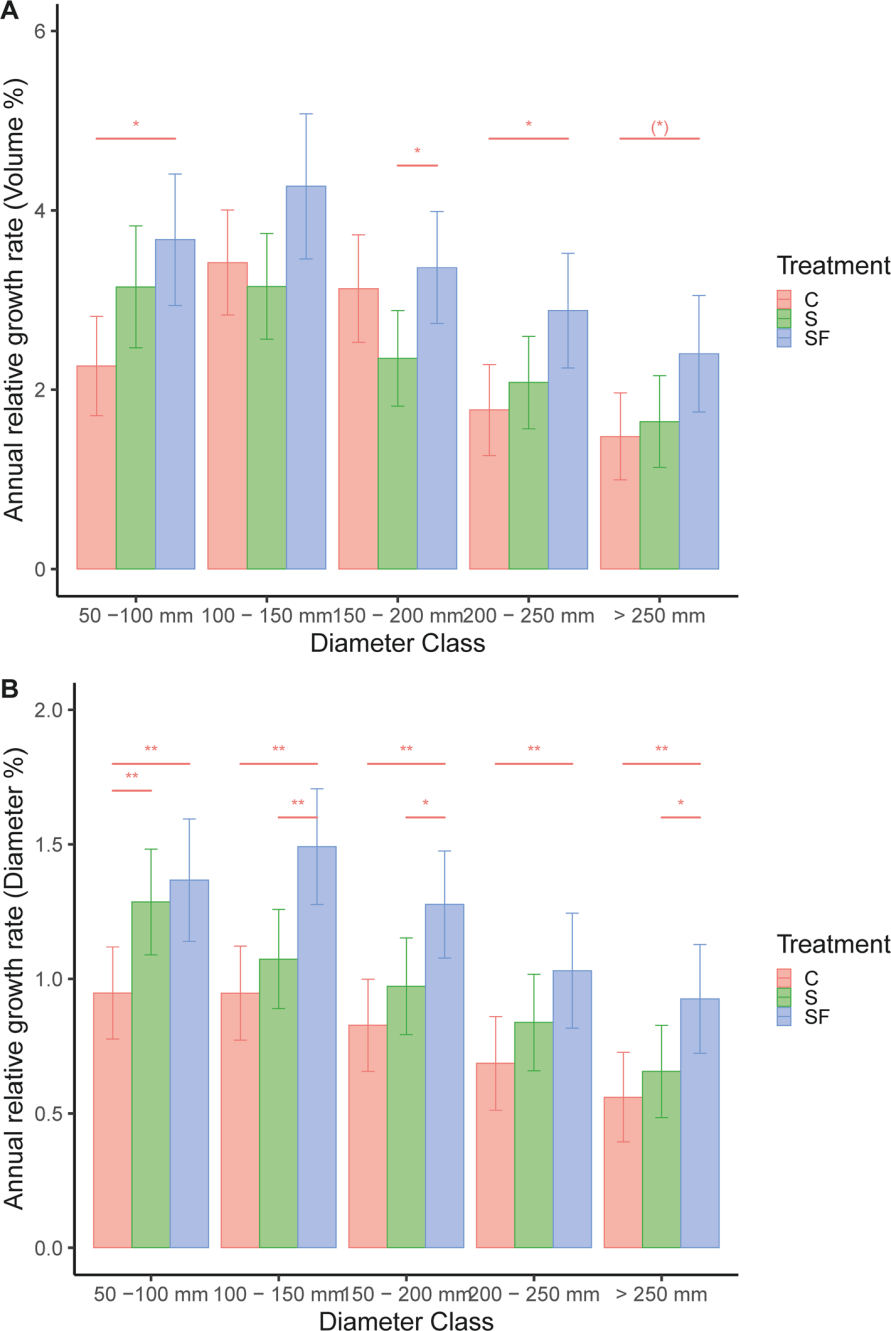
The relative tree-specific annual volume growth (**A**) and diameter (**B**) growth of five different size classes (dbh = 50–100, 100–150, 150–200, > 250 mm) within three treatments (*C* control, *S* single-tree selection, *SF* single-tree selection with fertilization). Shown are least square means with 95% confidence intervals. Within each diameter class, pairwise significant differences are denoted with an asterisk on a horizontal line (*p = 0.05–0.10, *p = 0.01–0.05, **p < 0.01)

At the tree level, growth differed among treatments (Volume: *F* = _2, 17_ = 5.68, *p* = 0.01; Diameter: *F*_2, 17_ = 13.3, *p* < 0.01; Fig. 3A, B). Growth also differed between diameter classes (Volume: *F*_4, 2792_ = 34.47, *p* < 0.01; Diameter: *F*_4, 2792_ = 35.3, *p* < 0.01; Fig. 3A, B), but the response depended on treatment (interaction Treatment x Growth; Volume: F_8, 2792_ = 3.57, *p* < 0.01; Diameter: *F*_8, 2792_ = 2.3, *p* = 0.02; Fig. 3A, B). A post hoc Bonferroni test revealed that tree volume growth was higher in the fertilized treatment within some diameter classes, whereas diameter growth was highest in the single-tree selection with fertilization (Fig. 3; Table S3).

#### Hypothesis 2: Seedlings and small trees

We found no evidence that the number *P. abies* seedlings differed among treatments, although the number of small trees decreased in the fertilized treatment (Tables 1, S4; Fig. S2). Seedling diversity displayed a near-significant interaction between treatment and time but the post hoc test revealed no difference between the before and after treatment (Tables 1, S4; Fig. S2).

**Table 1:** The influence of treatment (Single-tree selection, Single-tree selection with fertilization, Control), time (before and after logging), and the interaction on seedling diversity, number of *P. abies* seedlings (dbh between 0.5 and 4.9 cm) and small trees (dbh = 0.5–4.9 cm). Shown are results from an ANOVA type 3

Response	Treatment			Time			Treatment × time		
	Df	*F*	*p*	Df	*F*	*p*	Df	*F*	*p*
Seedling diversity	2, 12.95	1.58	0.24	1, 19	0.71	0.41	2, 19	2.84	0.08
*P. abies*seedlings	2, 12.18	3.10	0.08	1, 19	20.70	** < 0.01**	2, 19	1.88	0.18
*P. abies*small trees	2, 15.16	0.07	0.94	1, 25	7.44	**0.01**	2, 25	3.02	0.07

Significant*p*-values are shown in bold

### Biodiversity

#### Hypothesis 3: Vascular plants and bryophytes

**Fig. 4:**
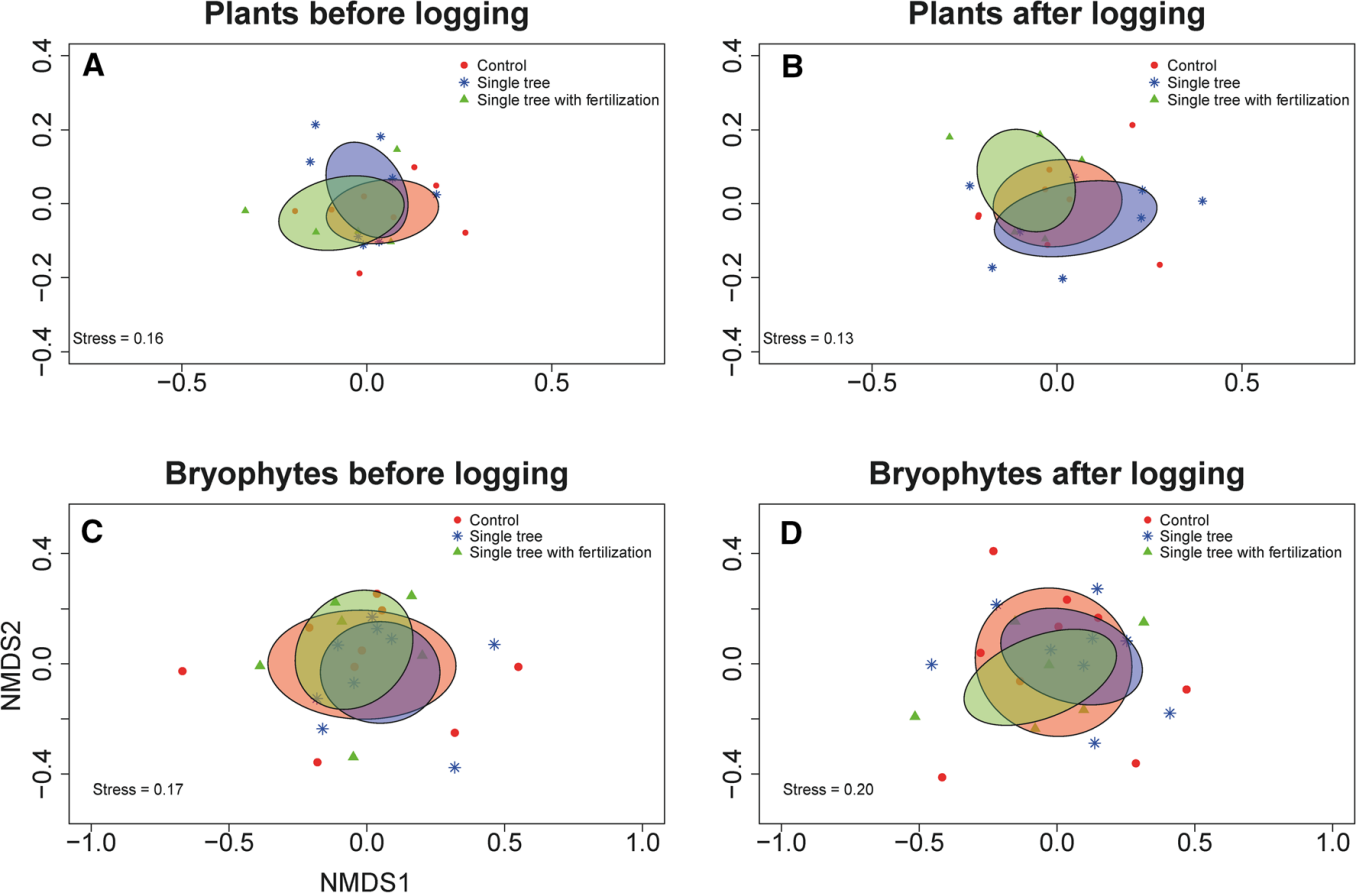
Two-dimensional NMDS plots displaying the community assemblage on abundance of plants (**A**, **B**) and the presence/absence of bryophytes (**C**, **D**), before (**A**, **C**), and after (**B**, **D**) logging treatment. Each community is displayed with a point. Treatment-specific ellipses represent the standard deviation calculated from the distance of each point to the centroid

We found no effect of logging on species assemblages of vascular plants (*F*_2, 36_ = 0. 65, *p* = 0.80; main effect Year: *F*_1, 36_ = 2.72, *p* = 0.02 main effect of Treatment: *F*_2, 36_ = 1.36, *p* = 0.20; Fig. 4A, B) or bryophytes (*F*_2, 42_ = 0.36, *p* = 0.99; main effect Year: *F*_1, 42_ = 3.98, *p* < 0.01 main effect of Treatment: *F*_2, 42_ = 0.74, *p* = 0.77; Figs. 4C, D, S3) as evident by the non-significant interaction between treatment and time in the PERMANOVA analysis. Neither did we detect any effect of single-tree selection or fertilization on species richness of vascular plants or bryophytes (Tables 2, 3). Two out of the five functional groups of bryophytes displayed a near-significant or significant interaction between treatment and time: Richness of liverwort species was lower after single-tree selection with a similar tendency (near-significant) in the control, whereas bryophytes associated with partly shaded and well-lit environments were lower in the control after treatment (Table 3, S4; Fig. S4).

**Table 2:** The influence of treatment (Single-tree selection, Single-tree selection with fertilization, Control), time (before and after logging), and the interaction on species richness of vascular plants, number of unoccupied (absent) points in the field layer and bottom layer and the abundance of dominant plant and bryophyte species. Shown are results from a marginal or type 3 ANOVA

Response	Treatment			Time			Treatment × time		
	Df	*F*	*p*	Df	*F*	*p*	Df	*F*	*p*
*Plants*									
Species richness	2, 11.32	1.10	0.37	1, 18	0.83	0.38	2, 18	0.6	0.56
*Vaccinium myrtillus*	2, 11.59	2.39	0.14	1, 18	9.66	**0.01**	2, 18	0.26	0.78
*Avenella flexuosa*	2, 11.48	2.59	0.12	1, 18	3.66	0.07	2, 18	7.51	** < 0.01**
*Vaccinium vitis-idaea*	2, 11.88	0.72	0.51	1, 18	1.94	0.18	2, 18	0.26	0.77
*Field layer absent*	2, 11.90	0.01	0.99	1, 18	15.57	** < 0.01**	2, 18	3.80	**0.04**
*Bryophytes*									
*Hylocomium splendens*	2, 11.69	0.62	0.56	1, 18	8.80	**0.01**	2, 18	19.43	** < 0.01**
*Pleurozium schreberi*	2, 11.33	1.26	0.32	1, 18	1.76	0.20	2, 18	0.01	0.99
*Ptilium crista-castrensis*	2, 11	0.25	0.78	1, 18	0.01	0.91	2, 18	0.55	0.59
*Sphagnum spp.*	2, 11.35	0.31	0.74	1, 18	4.61	**0.05**	2, 18	1.90	0.18
*Dicranum spp.*	2, 11.47	3.91	**0.05**	1, 18	3.86	0.07	2, 18	0.40	0.67
*Bottom layer absent*	2, 11.26	5.65	**0.02**	1, 18	41.64	** < 0.01**	2, 18	11.85	** < 0.01**

Significant* p*-values are shown in bold

**Table 3:** The influence of treatment (Single-tree selection, Single-tree selection with fertilization, Control), time (before and after logging), and the interaction on the total number of bryophyte species, number of species in four functional groups of bryophytes and for number of liverwort species. Shown are results from an ANOVA type 3

Functional groups	Treatment			Time			Treatment × time		
	Df	*F*	*p*	Df	*F*	*p*	Df	*F*	*p*
Species richness	2, 13.50	0.04	0.96	1, 21	7.40	**0.01**	2, 21	1.31	0.29
Closed forest	2, 13.55	1.06	0.37	1, 21	11.25	** < 0.01**	2, 21	1.15	0.34
Forest and open land	2, 13.67	0.13	0.88	1, 21	4.60	**0.04**	2, 21	0.02	0.98
Shade and semi-shade	2, 13.45	1.19	0.34	1, 21	1.49	0.24	2, 21	0.77	0.48
Partly shaded and well-lit	2, 13.61	0.05	0.95	1, 21	5.44	**0.03**	2, 21	4.38	**0.03**
Liverworts	2, 13.25	0.24	0.79	1, 21	26.54	** < 0.01**	2, 21	2.78	0.08

Significant* p*-values are shown in bold

**Fig. 5:**
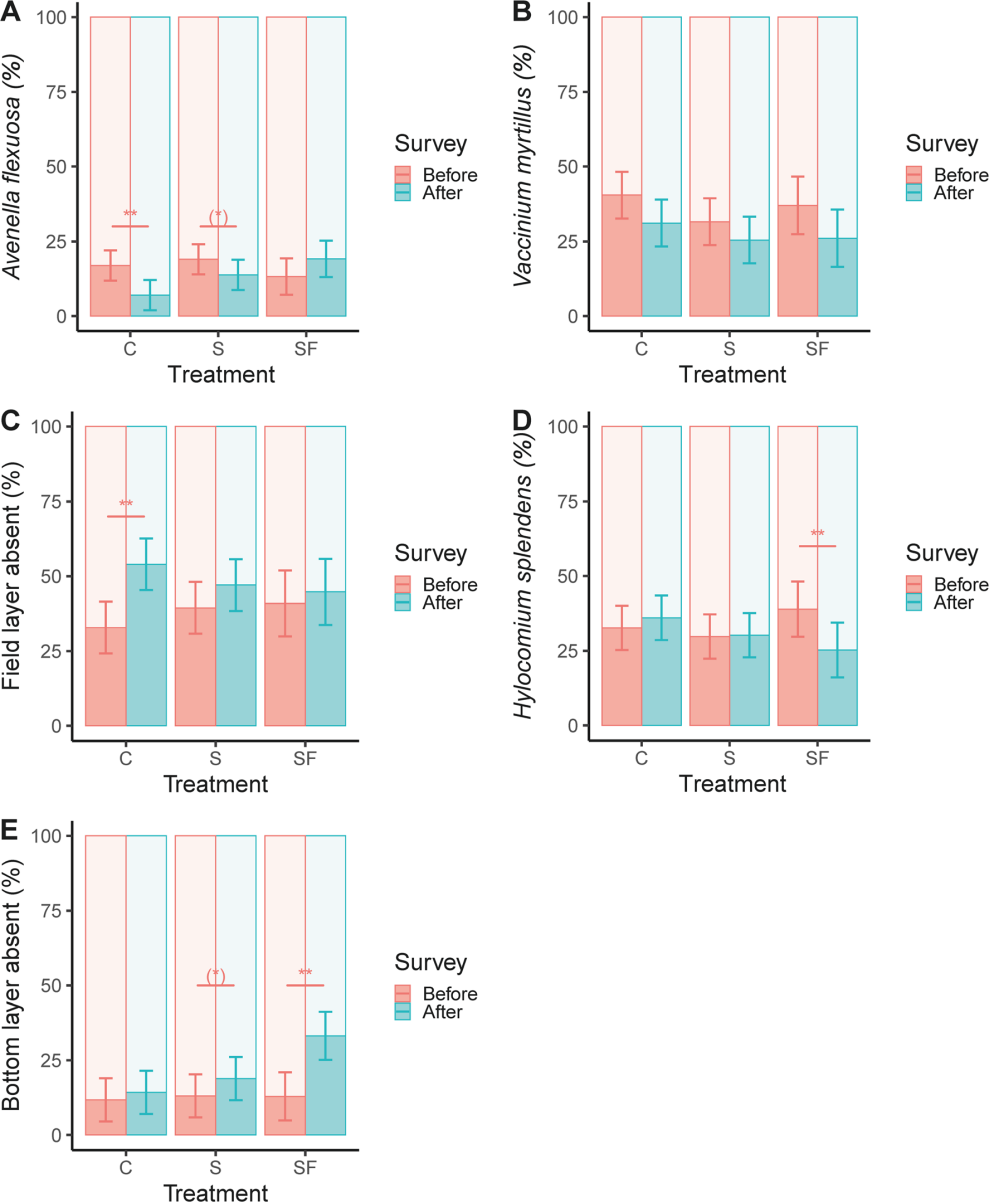
The percent of points (386–400 points surveyed per plot) occupied in the field layer by *A. flexuosa* (**A**), *V. myrtillus* (**B**), percent of points in the field layer not occupied by vascular plants (**C**), percent of *H. splendens* in the bottom layer (**D**) and percent of points not occupied in the bottom layer (**E**), before and after each logging treatment (*C* control, *S* single-tree selection, *SF* single-tree selection with fertilization). Shown are least square means with 95% confidence intervals. For species that display a significant interaction in Table 2, a post hoc test reveals differences before and after each treatment (denoted with an asterisk on a horizontal line *p = 0.05–0.10, *p = 0.01–0.05, **p < 0.01)

At the species level, the abundance in two out of eight species was influenced by treatment as evident by a significant interaction (Figs. 5, S5): The grass species *Avenella flexuosa* (L.) Trin. was reduced in the control treatment after fertilization (Fig. 5A; Tables 2, S4), whereas the moss species *Hylocomium splendens* (Hedw.) Schimp. was less abundant in the fertilized treatment (Fig. 5D; Tables 2, S4). We found no evidence of a treatment effect on *Vaccinium myrtillus* L. In the field layer, the number of occupied points in the control was lower after treatment. The number of occupied points in the bottom layer was lower after fertilization, with a similar (non-significant) tendency after single-tree selection (Fig. 5C, E; Tables 2, S4).

#### Hypothesis 4: Wood-inhabiting fungi

**Fig. 6:**
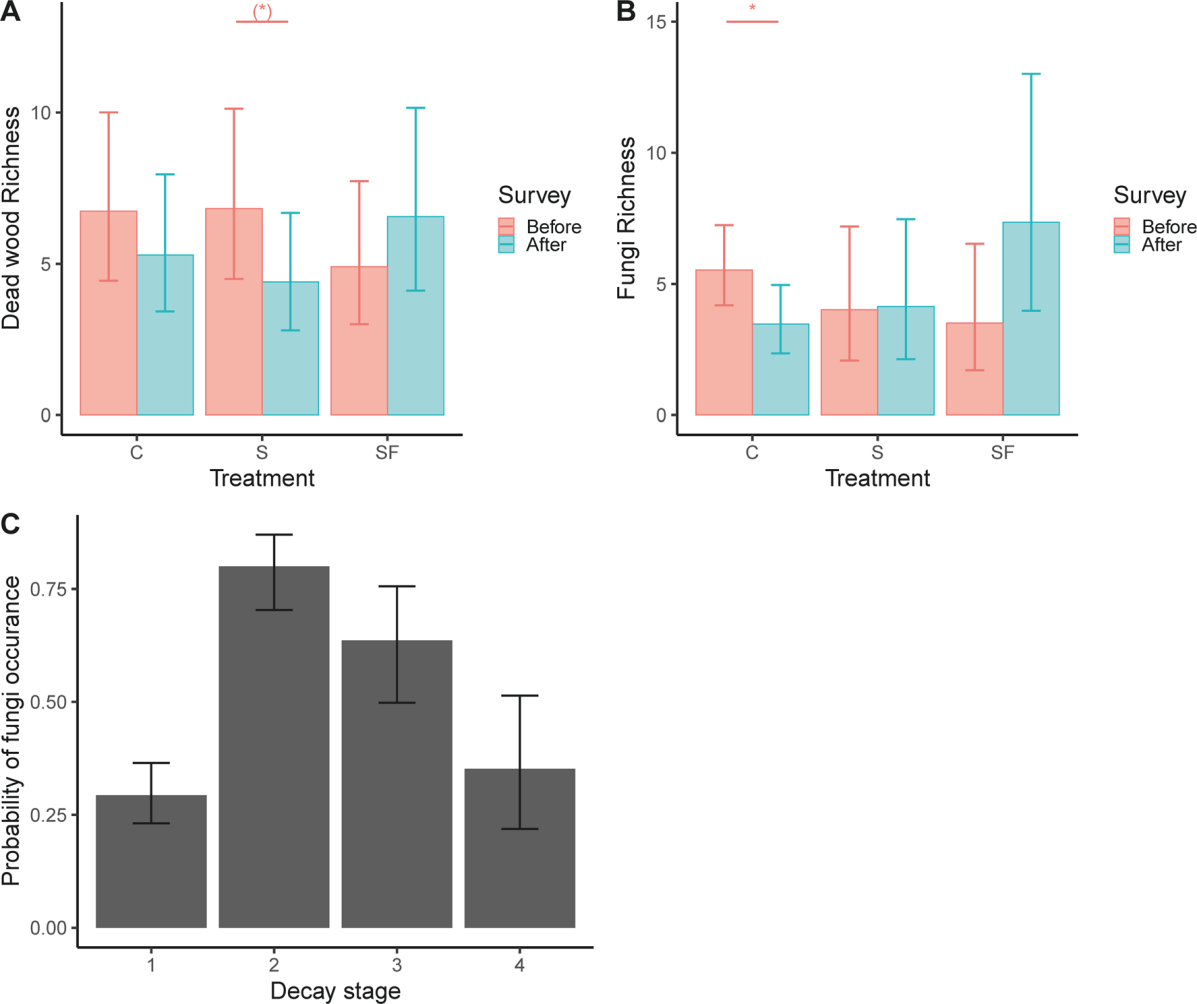
Dead wood richness (**A**), species richness of wood-inhabiting fungi (**B**) before and after each logging treatment (*C* control, *S* single-tree selection, *SF* single-tree selection with fertilization), and the probability of wood-inhabiting fungi infestation on DW objects in different decay classes (**C**) (1 = Hard, 2 = Partly decayed, 3 = decayed, 4 = Much decayed). Shown are least square means with 95% confidence intervals, where figure **A**, **B** are back-transformed from a log + 1 − transformation and **C** from a logit-transformation. For **A** and **B**, a post hoc test reveals differences before and after each treatment (denoted with an asterisk on a horizontal line *p = 0.05–0.10, *p = 0.01–0.05, **p < 0.01)

DW richness displayed a significant interaction between treatment and time, where DW richness tended to be lower in the treatment subjected to single-tree selection (Fig. 6; Tables 4, S5). We found no effect of treatment on DW volume (Table 4). Species richness of wood-inhabiting fungi had a significant interaction between treatment and time, with a lower number of species in the control after than before the treatment (Fig. 6; Table 4, S5). Probability of fungi infestation did not differ among treatments, but infestation varied among decay stages. Both probability of infestation and species richness of wood-inhabiting fungi was influenced by dead wood volume (Table 4).

**Table 4:** The influence of treatment (Single-tree selection, Single-tree selection with fertilization, Control), time (before and after logging) and the interaction on dead wood richness, dead wood volume, probability of wood-inhabiting fungi infestation, and species richness of wood-inhabiting fungi. Shown is also dead wood volume which was included as a covariate in the analysis of species richness of wood-inhabiting fungi and probability of dead wood infestation. Decay stage was also included as an explanatory variable for probability of dead wood infestation. Shown are results from a marginal or type 3 ANOVA

Response	Treatment			Time			Treatment × time			Dead wood volume			Decay stage		
	Df	*χ*^2^/*F*	*p*	Df	*χ*^2^/*F*	*p*	Df	*χ*^2^/*F*	*p*	Df	*χ*^2^/*F*	*p*	Df	*χ*^2^/*F*	*p*
Dead wood richness	2, 13.26	0.19	0.83	1, 21	1.42	0.25	2, 21	3.55	**0.05**	–	–	–	–	–	–
Dead wood volume	2, 13.31	0.94	0.42	1, 21	4.42	**0.05**	2, 21	1.53	0.24	–	–	–	–	–	–
Infested dead wood*	2	3.08	0.21	1	4.53	**0.03**	2	0.21	0.90	2	17.49	** < 0.01**	3	87.89	** < 0.01**
Wood-inhabiting fungi Richness*	2, 21	3.00	0.07	1, 19	8.00	**0.01**	2, 19	4.41	**0.03**	2, 19	46.59	** < 0.01**	–	–	–

Significant* p*-values are shown in bold

*Site was excluded as a random effect due to model singularity

## Discussion

In this study, we have examined the short-term effect of single-tree selection on biodiversity and yield within the same experimental plots. We found that growth on both the plot level and tree level was higher in the single-tree selection and fertilized treatment, than in the single-tree selection and control (most pronounced in diameter and basal area growth). However, single-tree selection came with an imprint on the plant community: (i) abundance of *A. flexuosa* was reduced in the control while other treatments were unchanged, suggesting that single-tree selection with and without fertilization benefit this species, (ii) *H. splendens* decreased in abundance after single-tree selection with fertilization, suggesting that fertilization do not favor this species. Yet, we found no evidence of a treatment-induced shift in abundance of the keystone species *V. myrtillus*. Species richness of wood-inhabiting fungi was higher after single-tree selection. As expected, wood-inhabiting fungi were also highly influenced by dead wood availability and decomposition stage.

### Hypothesis 1: Single-tree selection and fertilization improved tree growth

Our prediction that single-tree selection and fertilization improved tree growth was supported since growth increased in the fertilized plots but not in unfertilized plots. The annual growth was 5.75 m^3^ ha^−1^ a^−1^ in the stand subjected to single-tree selection with fertilization, which was 37% (4.20 m^3^ ha^−1^ a^−1^) and 27% (4.52 m^3^ ha^−1^ a^−1^) higher than in the single-tree selection and control plot (the latter not significant), respectively. Given the large body of literature in the rotation forestry system that have found an increase in yield after nitrogen fertilization and that forests in northern Sweden have a high C/N ratio (Högberg et al. 2021), the increase in growth is expected. If fertilization is applied on a regular basis over ten years, it can increase annual growth by four times in northern Sweden (Bergh et al. 1999). Fertilization could also increase growth substantially on the national level (Bergh et al. 2005). Whether such long-term effects are possible to achieve within selection system is not known, but fertilization offers a promising tool to increase the short-term production after single-tree selection.

Individual tree growth after single-tree selection with and without fertilization was almost always higher in relation to the control, but this difference was only significant in the fertilized treatment. This is consistent with the studies in the rotation forestry system, where thinning does not increase stand-level growth, but the remaining trees respond to the open environment by a diameter increment (Mäkinen and Isomäki 2004; Figs. 2, 3). In contrast to the rotation forestry system where the most vigorous trees are favored by thinning from below, trees remaining after single-tree selection are co-dominant and less vigorous trees which could be slow at responding to the partial release in light. Hynynen et al. (2019) found that the basal area growth in even-aged stands after thinning was faster than uneven-aged stands, which was explained by a time lag in growth for suppressed trees. In contrast, Valkonen et al. (2017) found a growth response already five years after a heavy diameter-limit cut, but the maximum growth was attained after ten years. Thus, the short-term growth response found here is likely to be accelerated in the future as suppressed trees are responding to the partial release in light.

### Hypothesis 2: Fertilization increased growth of small trees

The number of seedlings and small trees is mainly driven by three processes: recruitment of new seedlings or small trees, growth of seedlings into the next size class and mortality (Lundqvist 1995). Contrary to our hypothesis, we found that the number *P. abies* seedlings was not influenced by single-tree selection or fertilization, whereas the number of small trees was in fact reduced in the fertilized treatment. A reduction of small trees in the fertilized could mean two things: either the mortality was high after fertilization or the small trees grew out of the size class, into the tree layer. Given that fertilization have an effect on tree growth (Bergh et al. 1999), we believe that the latter is the most likely scenario. To fully disentangle the dynamics of small trees and seedlings the process of recruitment, mortality and growth need to be followed individually. But here we find evidence that fertilization also influences the growth of smaller trees.

### Hypothesis 3: Fertilization left an imprint on plant and bryophyte abundance

Our hypothesis that single-tree selection would leave a small imprint on the vascular plant and bryophyte assemblage by favoring nutrient-sensitive and light-sensitive species was partly supported. At the level of species assemblages of vascular plants (measured as abundance) and bryophytes (measured as presence/absence), we found no effect of single-tree selection or fertilization. This is in contrast to the turnover in species communities after clear-cutting, but in line with the previous studies on effects of single-tree selection on fungal and beetle communities (Joelsson et al. 2017, 2018; Kim et al. 2021). However, a reduction in number of bryophyte species associated with open environments in the control but not in other treatments (Fig. S4A) suggests that the more open environment associated with single-tree selection favored these species. The pattern for liverwort species was not as clear: number of species became fewer after single-tree selection, but a near-significant effect was also apparent in the control treatment. Notably, our results on the assemblage level only cover the short-term responses of vascular plants and bryophytes and the analysis of species richness is a rather course measure of biodiversity. However, previous studies have detected both a short-term (~ 1 year after logging) and long-term response of forest management using species richness (Jalonen and Vanha-Majamaa 2001; Fenton et al. 2003; Dynesius 2015). More fine-scaled differences could be detected if, for instance, the abundance of all bryophyte species had been recorded. But from this study, the relatively weak imprint of treatment on species richness and species assemblage suggest that there is no rapid turnover in the species community.

At the species level, a significant interaction between treatment and time was found for *A. flexuosa* and *H. splendens*. A post hoc test comparing abundance before and after treatment revealed that *A. flexuosa* and *H. splendens* was reduced in the control and fertilized treatment, respectively (Fig. 5A, D). It is well established that both nitrogen and light benefit *A. flexuosa* (Foggo 1989; Högbom and Högberg 1991) and studies have found that both fertilization and clear-cutting promote *A. flexuosa* (Strengbom et al. 2001, 2018; Palviainen et al. 2005; Strengbom and Nordin 2008). Therefore, we interpret the reduction of *A. flexuosa* in the unharvested control, but not in the two other treatments, as single-tree selection benefitting *A. flexuosa*. We would also have expected a higher abundance in the fertilized treatment, but this effect was not significant. The fact that we only detected a reduction in the control could be due to several factors such as annual population fluctuations, long-term negative population trends mitigated by single-tree selection or detection bias from field staff (a different set of staff did the before and after survey).

A reduction in the abundance of the bryophyte *H. splendens* after fertilization is an expected result. In comparison to other bryophyte species, *H. splendens* growth is particularly sensitive to high nitrogen levels (Salemaa et al. 2008) and both fertilization and clear-cutting have negative effect on this species (Strengbom et al. 2001, 2018; Palviainen et al. 2005).

We did not find an effect of treatment on the keystone species *V. myrtillus*, which has recently decreased in abundance in the boreal region of Sweden (Hedwall et al. 2013). This species is utilized by a wide range of organisms such as insects, ungulates, and brown bears, but is also a common plant for berry picking (Atlegrim 1989; Vaara et al. 2013; Hertel et al. 2016; Spitzer et al. 2021). It is negatively affected by logging, but then gradually recovers (Atlegrim and Sjöberg 1996; Bergstedt and Milberg 2001; Hedwall et al. 2013). In contrast to our findings, previous studies found a reduction in abundance after selective logging (Atlegrim and Sjöberg 1996; Vanha-Majamaa et al. 2017). Atlegrim and Sjöberg (1996) found a patchier distribution of *V. myrtillus* after logging but no effect on shoot survival. However, the latter study removed a higher proportion of the volume (40–50%) and in Vanha-Majamaa et al. (2017) the pre-treatment abundance was used as a control instead of an additional control plot. In our study, we found a reduction in *V. myrtillus* after logging but this was also observed in the control plot. Thus, our results suggest that single-tree selection is able to maintain the cover of *V. myrtillus*.

In this experiment, we only investigated the short-term effect of fertilization on biodiversity and found small effects on two plant species. However, the effects can be long-lasting (Strengbom and Nordin 2008) and more excelled after repeated fertilization. Intensive fertilization in the rotation forestry system results in a dense canopy cover, low structural heterogeneity, shortened rotation time, and low amount of dead wood which can have a negative impact on biodiversity (Strengbom et al. 2011). As the fertilization effect is larger in northern Sweden than southern Sweden, at least when considering tree growth (Bergh et al. 1999), it is plausible that local effects on biodiversity could differ depending on latitude. For instance, fertilization at northern latitudes could result in a larger relative response in canopy closure, resulting in less light available for understory species, relative to unfertilized stands. On the other hand, biodiversity might be favored by the structural heterogeneity and stable environment associated with selection system. Thus, it is difficult to assess the potential long-term consequences of fertilization in the selection system on biodiversity. This needs to be further investigated.

### Hypothesis 4: Wood-inhabiting fungi are influenced by dead wood availability and decay stage

We hypothesized that any effect of treatment on wood-inhabiting fungi would come from treatment effects on dead wood availability and not treatment per se. In partial support of this hypothesis, we found a near-significant effect of lower DW richness after single-tree selection and a lower richness of wood-inhabiting fungi in the control after the second survey. Although the causes of these effects should be interpreted with care, we highlight that the trend in DW richness was positive from fertilization but negative in both control and single-tree selection, and that this increase co-occurred with an increase in richness of wood fungi. Previous studies have proposed that both nitrogen addition and higher temperatures increase decomposition rates of dead wood (van der Wal et al. 2007; Finér et al. 2016). It is possible that fertilization in combination with a warmer microclimate after logging stimulated the fungal activity and the production of fruiting bodies, resulting in a high richness of DW (DW in several decay stages) and many fruiting bodies. However, we leave it for future studies to disentangle the mechanisms behind the observed pattern. Furthermore, we also found that DW volume to be a good predictor of the number of wood-inhabiting fungi species but this effect was irrespective of treatment. We also found that medium decay stages were more likely to be colonized, which usually also holds the most number of species (Junninen and Komonen 2011).

### Experimental limitations

We want to emphasize that species-specific responses in this study were only assessed for the most frequently occurring species. A few red-listed species were included in the analysis of fungi richness and bryophyte assemblage, but these are too few to draw any conclusion regarding the response of rare or threatened species. Furthermore, we also stress that the number of replicates in the fertilized treatment were lower than for the other treatments (*n *= 5 for vascular plants and bryophytes, *n* = 6 for wood-inhabiting fungi), which slightly reduce the power for detecting effects of fertilization. Finally, this study covers the short-term response of biodiversity and tree growth to single-tree selection. It remains to be investigated whether the patterns observed here are consistent through time.

## Conclusion

Most, if not all, studies examining the effects of continuous cover forestry have focused on either production or biodiversity and hence failed to address the potential conflicting goals that often are referred to when discussing this management alternative. Here, we simultaneously examined the initial and short-term response of both forest biomass production and biodiversity to single-tree selection within the same experimental setup using a before-after-control-impact study design. We show that single-tree selection with fertilization produced the highest yield and that species assemblages were intact after single-tree selection. Notably, the (one-time) application of nitrogen left an imprint on the abundance of one vascular plant and one bryophyte species. While this experiment evaluated the short-term effect of single-tree selection, it is more challenging to make long-term predictions. For instance, repeated fertilization will probably increase yield even more but potentially also come with pronounced effects on the plant community. Thus, it remains to be investigated whether the pattern observed here persists over several logging cycles and also how biodiversity compares to the rotation forestry system. Therefore, we hope that this work will stimulate new studies investigating the long-term effects of selection system on yield and biodiversity, but also how single-tree selection influences other taxonomic groups not addressed here, ecosystem services, and threatened species. But so far, we believe that single-tree selection, which is practiced within the selection system, is an interesting tool that might have the potential to reduce the conflict between forest production and biodiversity conservation.

## Supplementary Information

Below is the link to the electronic supplementary material.

Supplementary file1 (PDF 1341 kb)
