# Supplementary material for: Short-term effects of continuous cover forestry on forest biomass production and biodiversity: Applying single-tree selection in forests dominated by Picea abies
Source: Ambio. 2022 Jun 4;51(12):2478–95. doi: 10.1007/s13280-022-01749-5 (PMC9584012; doi:10.1007/s13280-022-01749-5)
Supplement: Supplementary file 1 — Supplementary file1 (PDF 1341 kb) [file 13280_2022_1749_MOESM1_ESM.pdf]

Ambio

Supplementary Information

This supplementary information has not been peer reviewed.

Title: Short-term effects of continuous cover forestry on forest biomass production and biodiversity - applying single-tree selection in forests dominated by *Picea abies*

Appendix S1 – Complementary figures and tables

Appendix S2 – Species list

Appendix S1 – Complementary figures and tables

Fig. S1. The frequency distribution before (A) and after (B) harvesting of all living trees with a dbh ≥ 5 cm divided into 8 size classes. Each subfigure represents a site and treatment combination (C= Control, S = Single-tree selection, SF= Single-tree selection with fertilization). Note that two sites of Ingatorp and Hjältanstorp had an extra replicate of single-tree selection (S2).

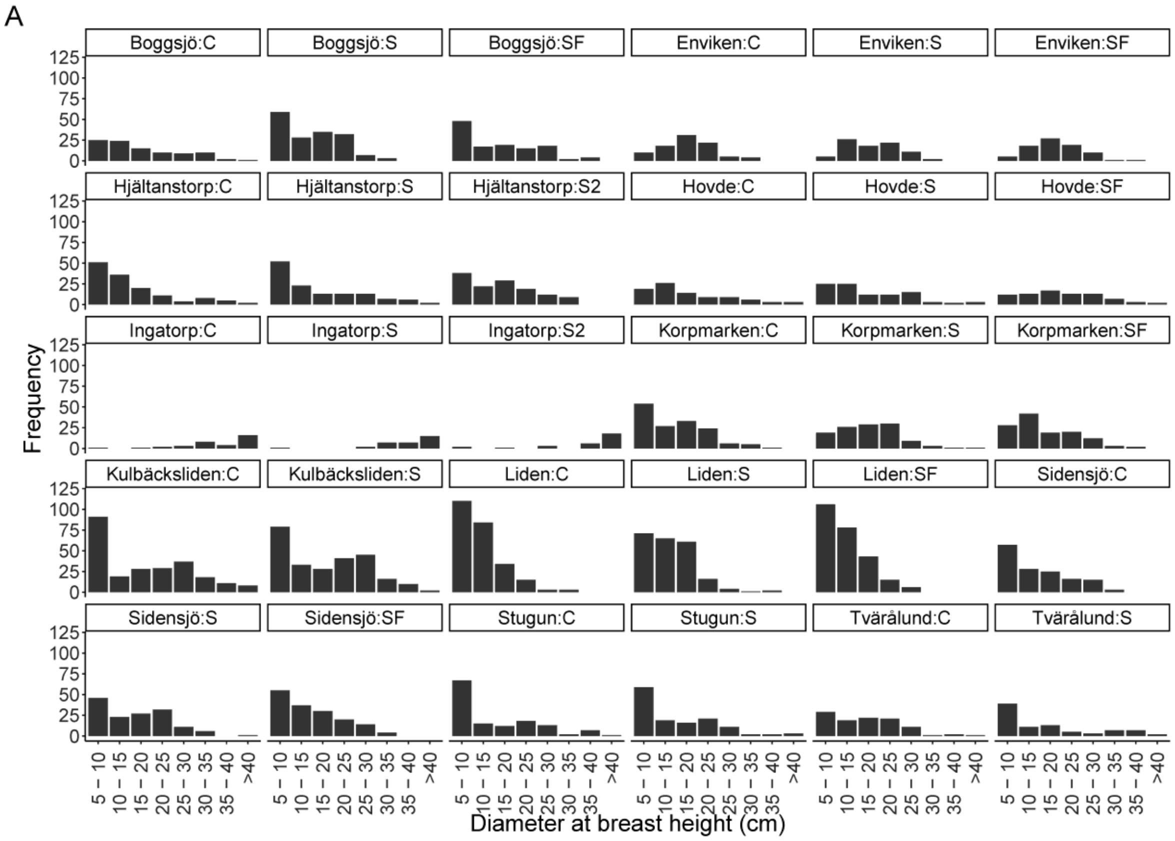

B

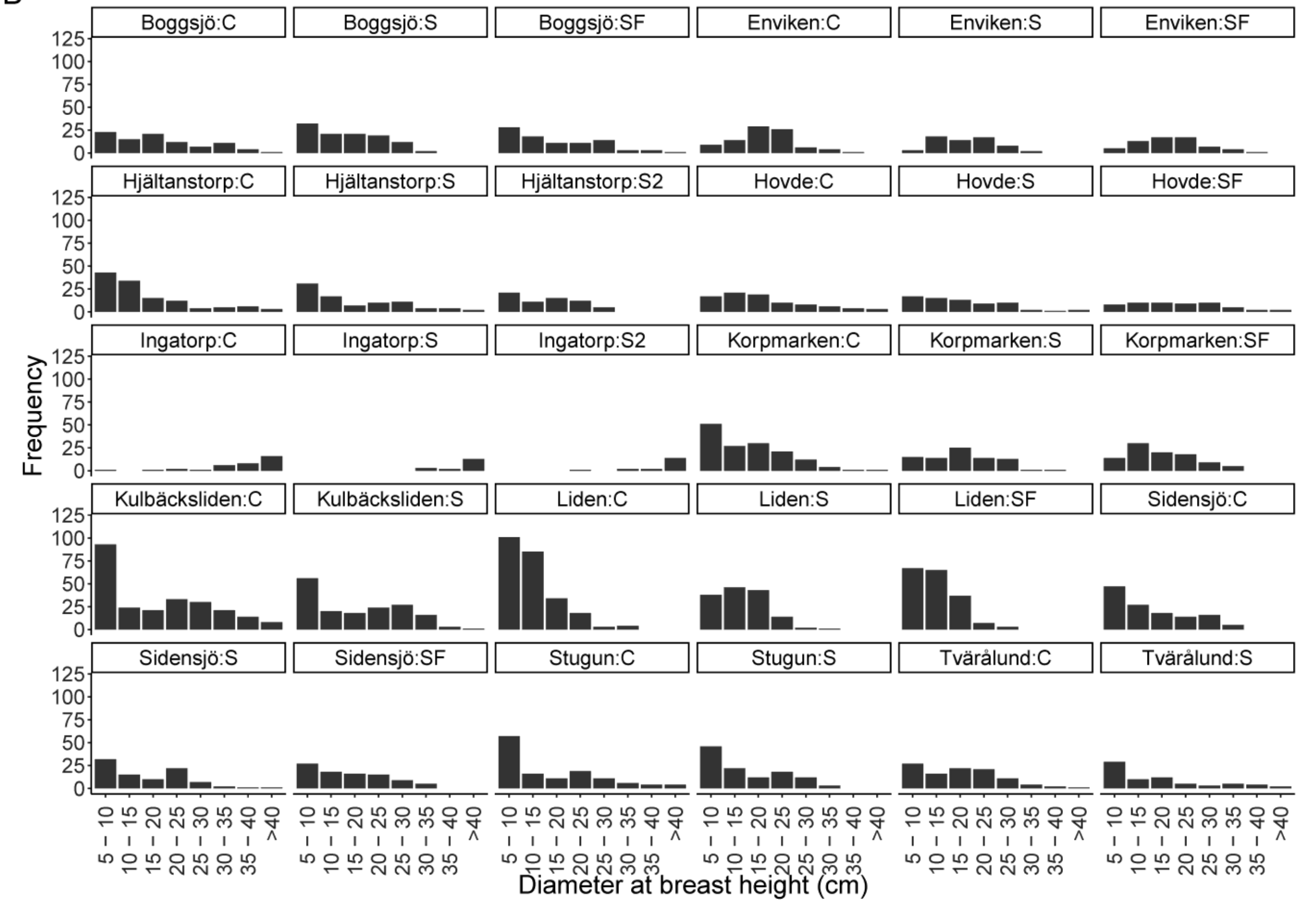
Boggsjö:C
Boggsjö:S
Boggsjö:SF
Enviken:C
Enviken:S
Enviken:SF
Hjältanstorp:C
Hjältanstorp:S
Hjältanstorp:S2
Hovde:C
Hovde:S
Hovde:SF
Ingatorp:C
Ingatorp:S
Ingatorp:S2
Korpmarken:C
Korpmarken:S
Korpmarken:SF
Kulbäcksliden:C
Kulbäcksliden:S
Liden:C
Liden:S
Liden:SF
Sidensjö:C
Sidensjö:S
Sidensjö:SF
Stugun:C
Stugun:S
Tväråliden:C
Tväråliden:S
Frequency
0
25
50
75
100
125
5 – 10
10 – 15
15 – 20
20 – 25
25 – 30
30 – 35
35 – 40
>40
Diameter at breast height (cm)

Fig. S2. The seedling (height 10 – 130 cm) diversity (A) and number of small trees of *P. abies* (dbh between 0.5 – 4.9 cm) measured over the whole 25 x 40 m plot (B), before and after each logging treatment (C= Control, S = Single-tree selection, SF= Single-tree selection with fertilization). Shown are least square means with 95% confidence intervals, small trees are back-transformed from a log + 1 - transformation. Within each treatment, pairwise significant differences are denoted with an asterisk on a horizontal line ((*) p = 0.05 - 0.10, * p = 0.01 - 0.05, ** p < 0.01).

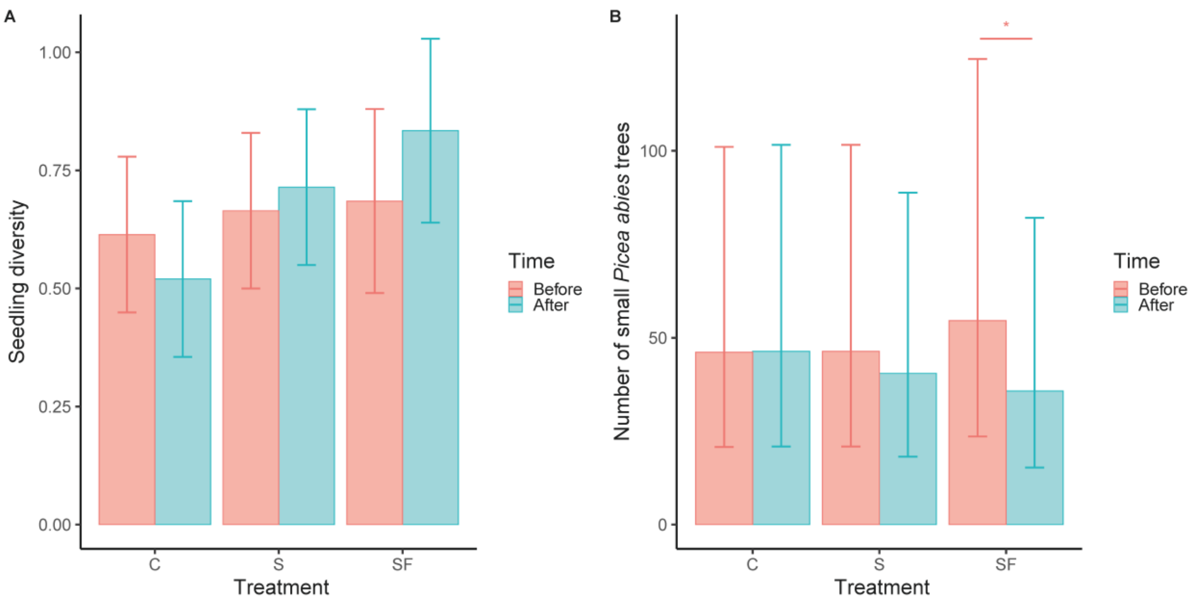

Fig. S3. Two-dimensional NMDS plots displaying the community composition of bryophytes species before (A, C, E) and after (B, D, F) logging. Each ellipse represents a logging treatment with the standard deviation calculated from the distance of each point to the centroid. Species are displayed in the background colored according to habitat preference (A, B; Forest vs. Forest and Open land), light sensitivity (C, D; Shade/Semis-hade vs. Partly shaded/well-lit) and taxonomic group (E, F; liverworts vs. other groups). See appendix S2 for the full species names.

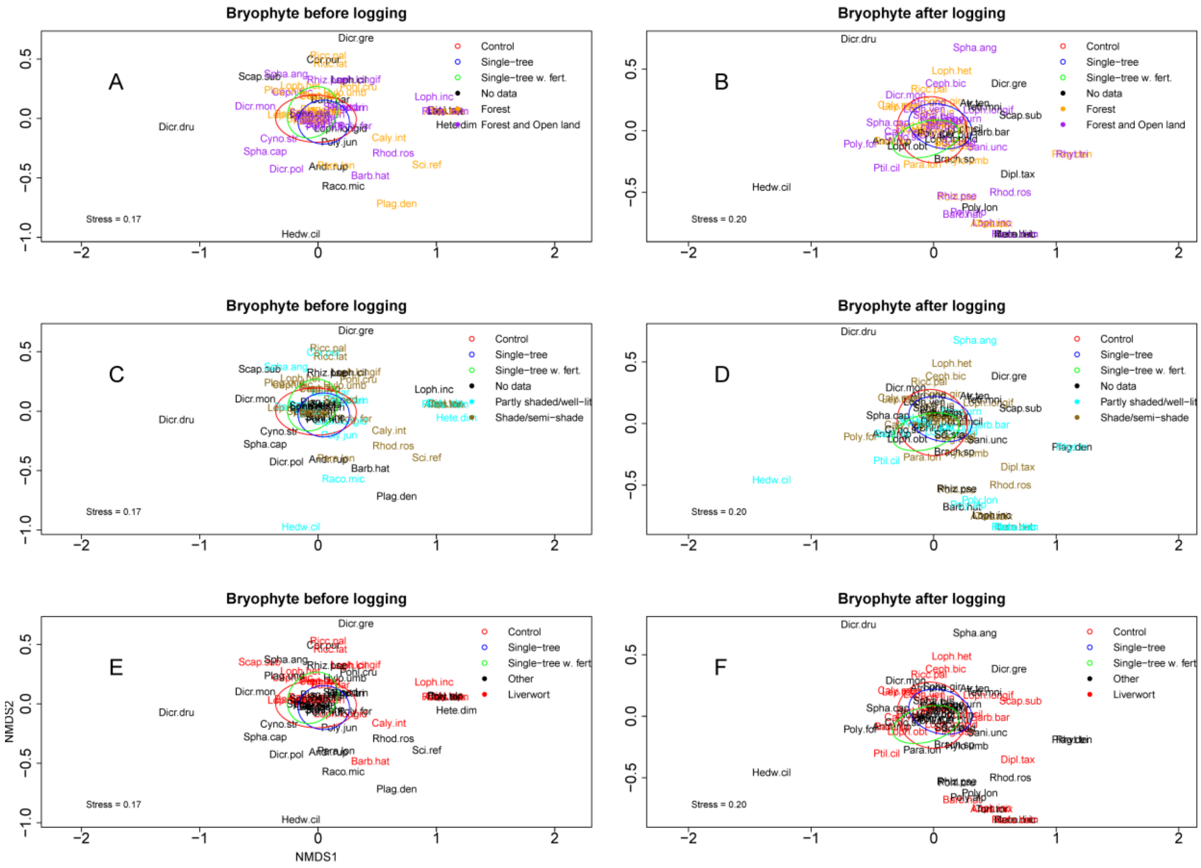

Fig. S4. The number of bryophyte species associated with partly shaded and well-lit environments (A) and number of liverwort species (B), before and after each logging treatment (C= Control, S = Single-tree selection, SF= Single-tree selection with fertilization). Shown are least square means with 95% confidence intervals, back-transformed from a log + 1 - transformation. Within each treatment, pairwise significant differences are denoted with an asterisk on a horizontal line ((*) p = 0.05 - 0.10, * p = 0.01 - 0.05, ** p < 0.01).

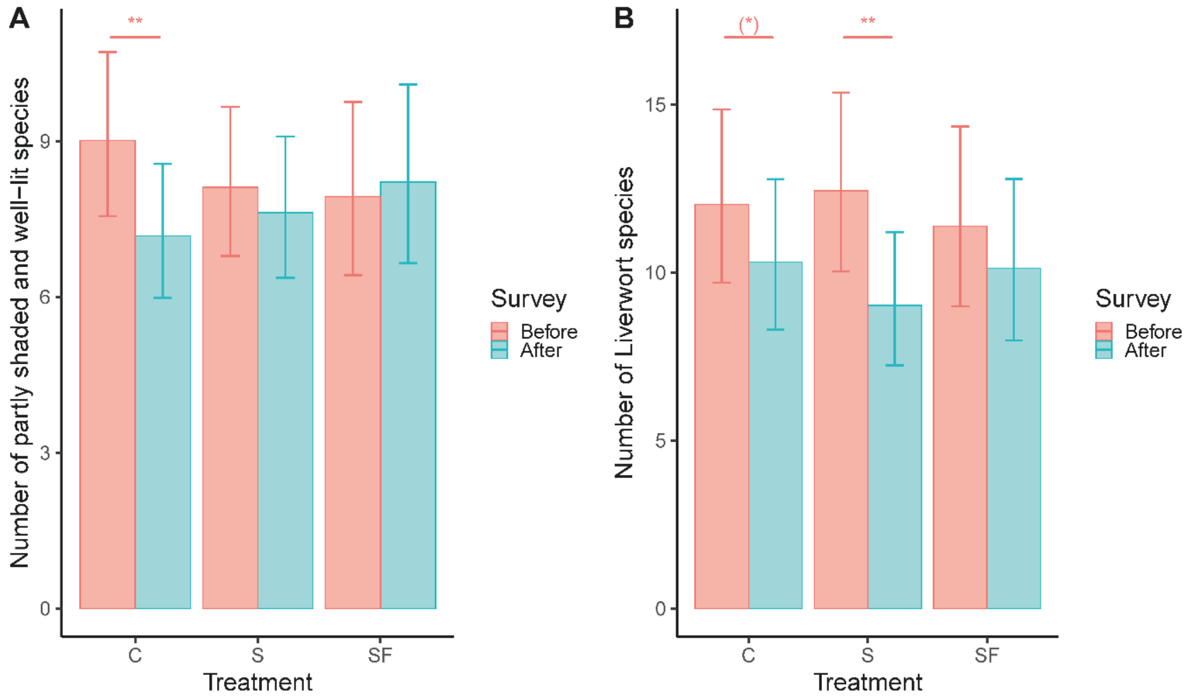

Fig. S5. The percent of points (386 – 400 points surveyed per plot) occupied in the field layer by *V. vitis-idaea* (A) and in the bottom layer by *P. crista-castrensis* (B), *P. schreberi* (C), *Sphagnum spp.* (D), *Dicranum spp.* (E), before and after each logging treatment (C= Control, S = Single-tree selection, SF= Single-tree selection with fertilization). Shown are least square means with 95% confidence intervals.

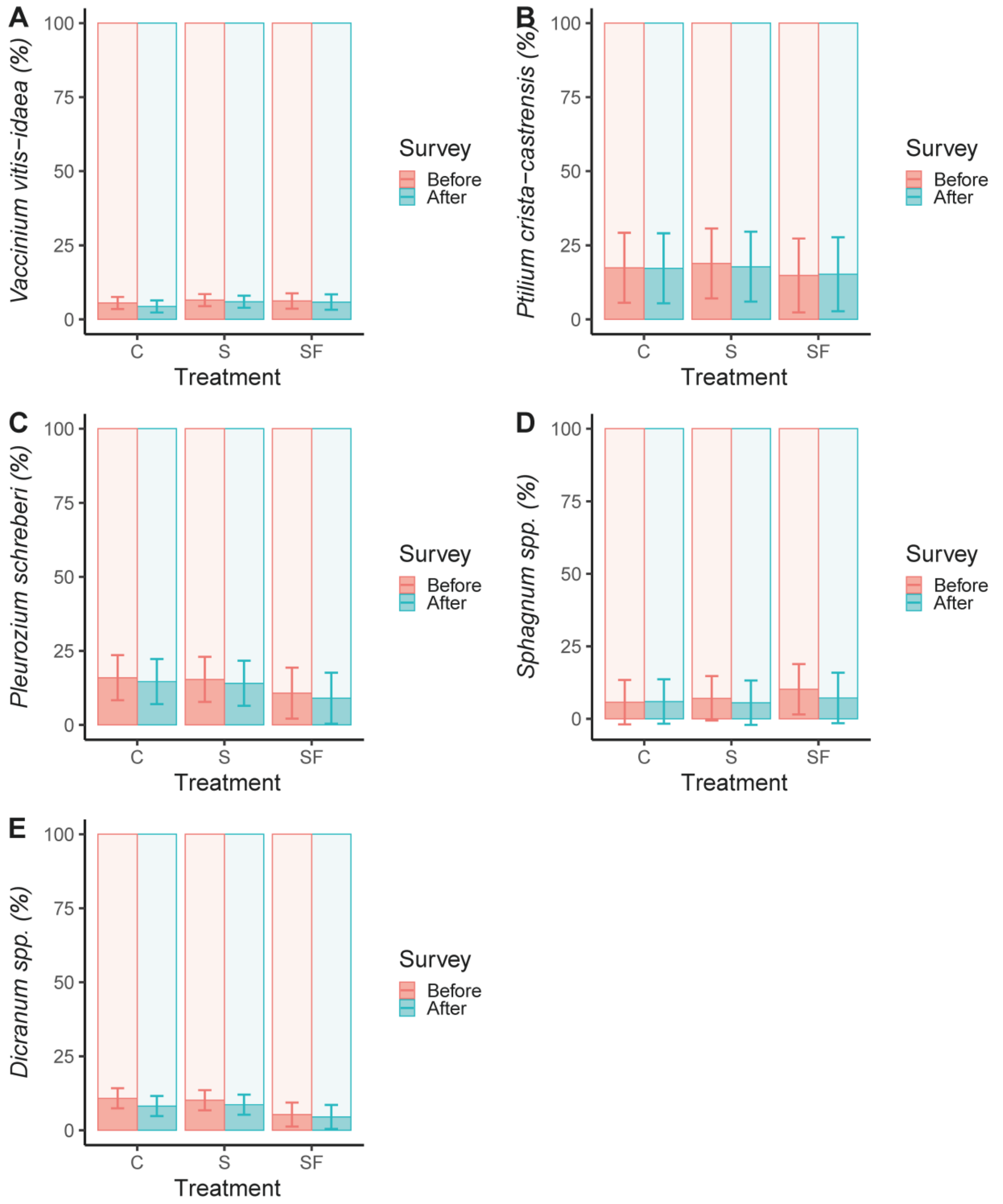

Table S1. The six types of surveys, described in the materials and methods section, which were done for each logging treatment (C= Control, S = Single-tree selection, SF= Single-tree selection with fertilization) across all eleven study sites.

Site	Treatment	Survey					
		Tree growth	Seedlings	Young trees	Bottom and Field layer	Bryophyte assemblage	Wood-inhabiting fungi
Boggsjö	C	x	x	x	x	x	x
Boggsjö	S	x	x	x	x	x	x
Boggsjö	SF	x	x	x	x	x	x
Enviken	C	x	x	x	x	x	x
Enviken	S	x	x	x	x	x	x
Enviken	SF	x	x	x	x	x	x
Hjältanstorp	C	x	x	x	x	x	x
Hjältanstorp	S	x	x	x	x	x	x
Hjältanstorp	S	x		x			
Hovde	C	x	x	x		x	x
Hovde	S	x	x	x		x	x
Hovde	SF	x	x	x		x	x
Ingatorp	C	x		x			
Ingatorp	S	x		x			
Ingatorp	S	x		x			
Korpmarken	C	x	x	x	x	x	x
Korpmarken	S	x	x	x	x	x	x
Korpmarken	SF	x	x	x	x	x	x
Kulbäcksliden	C	x		x	x	x	x
Kulbäcksliden	S	x		x	x	x	x
Liden	C	x	x	x	x	x	x
Liden	S	x	x	x	x	x	x
Liden	SF	x	x	x	x	x	x
Sidensjö	C	x	x	x	x	x	x
Sidensjö	S	x	x	x	x	x	x
Sidensjö	SF	x	x	x	x	x	x
Stugun	C	x		x			
Stugun	S	x		x			
Tväråland	C	x	x	x	x	x	x
Tväråland	S	x	x	x	x	x	x

Table S2. Test statistics from a post-hoc Bonferroni test on pairwise comparisons of plot-level annual growth (volume and basal area) and annual relative growth rate (volume) within three logging treatments. Test results are visualized by horizontal lines in fig. 2.

**Size class**	Control – Single-tree			Control - Single-tree and fert			Single-tree - Single-tree and fert		
	Df	t - ratio	*P*	Df	t - ratio	*P*	Df	t - ratio	*P*
Annual Growth (m3/ha)	16	0.49	1	16	-1.66	0.35	16	-4.02	**< 0.01**
Annual Growth (m2/ha)	20.1	-0.52	1	18.9	-3.94	**< 0.01**	16.8	-5.96	**< 0.01**
Annual relative volume growth rate (%)	17.1	-1.38	0.56	17.7	-5.09	**< 0.01**	18	-4.04	**< 0.01**

Table S3. Test statistics from a post-hoc Bonferroni test on the relative tree-specific annual volume and dbh growth of five size classes of *P. abies* subjected to three logging treatments. Each test shows pairwise comparisons between two treatments within a specific diameter class. Test results are visualized by horizontal lines in fig. 3.

**Size class**	Control – Single-tree			Control - Single-tree and fert			Single-tree - Single-tree and fert		
Volume	Df	t - ratio	*P*	Df	t - ratio	*P*	Df	t - ratio	*P*
50 – 100 mm	17	-2.85	0.17	17	-4.24	**0.01**	17	-1.39	1
100 – 150 mm	17	0.94	1	17	-2.26	0.55	17	-2.96	0.13
150 – 200 mm	17	2.92	0.14	17	-0.77	1	17	-3.65	**0.03**
200 – 250 mm	17	-1.42	1	17	-4.03	**0.01**	17	-2.89	0.15
> 250 mm	17	-0.82	1	17	-3.36	0.06	17	-2.63	0.26
Dbh									
50 – 100 mm	17	-4.86	**< 0.01**	17	-4.85	**< 0.01**	17	-0.84	1
100 – 150 mm	17	-1,96	0.99	17	-6.68	**< 0.01**	17	-4.89	**< 0.01**
150 – 200 mm	17	-2.41	0.41	17	-6.34	**< 0.01**	17	-4.05	**0.01**
200 – 250 mm	17	-2.48	0.36	17	-4.29	**< 0.01**	17	-2.32	0.49
> 250 mm	17	-1.87	1	17	-5.14	**< 0.01**	17	-3.62	**0.03**

Table S4. Test statistics from a post-hoc Bonferroni test on pairwise comparisons within each treatment, before and after treatment, for three analyses i) the percent of points (386 – 400 points surveyed per plot) occupied by *A. flexuosa* and *H. splendens,* and percent of points not occupied in field and ground layer, ii) number of species in functional groups of bryophytes, and iii) number of small *P. abies* (dbh between 0.5 – 4.9 cm) and seedling diversity. Test results are visualized by horizontal lines in fig. 5, fig. S2 and fig. S3.

**Response**	Control			Single-tree			Single-tree and fert		
	Df	t - ratio	*P*	Df	t - ratio	*P*	Df	t - ratio	*P*
**Points**									
Avenella flexuosa	18	-3.89	**< 0.01**	18	-2.05	0.16	18	1.83	0.25
Field layer absent	18	4.85	**< 0.01**	18	1.75	0.29	18	0.70	1
Hylocomium splendens	18	1.91	0.22	18	0.25	1	18	-6.16	**< 0.01**
Ground layer absent	18	1.08	0.89	18	2.48	0.07	18	6.87	**< 0.01**
**Bryophyte func. group**									
Partly shaded and well-lit	21	-3.9	**< 0.01**	21	-1.06	0.91	21	0.48	**1**
Liverwort	21	-2.50	0.06	21	-5.22	**< 0.01**	21	-1.57	0.40
**Seedlings and small trees**									
Number of small *P. abies* trees	25	0.05	1	25	-1.31	0.60	25	-3.01	**0.02**
Seedling diversity	19	-1.38	0.55	19	0.73	1	19	1.90	0.22

Table S5. Test statistics from a post-hoc Bonferroni test on pairwise comparisons within each treatment, before and after treatment, on the richness of dead wood (calculated as number of different combinations of dead wood size, type, decay stage and species) and species richness of polypore fruiting bodies. Test results are visualized by horizontal lines in fig. 6.

**Response**	Control			Single-tree			Single-tree and fert		
	Df	t - ratio	*P*	Df	t - ratio	*P*	Df	t - ratio	*P*
Dead wood Richness	21	-1.39	0.53	21	-2.50	0.06	21	1.35	0.57
Polypore Richness	32	-2.83	**0.03**	32	0.08	1	32	1.90	0.22

Appendix S2 – Species list

The species included in the biodiversity survey.

**Vascular plants**	**Wood-inhabiting fungi**	**Bryophytes**	**Bryophytes abbreviation**
Avenella flexuosa	*Antrodia heteromorpha*	*Crossocalyx hellerianus*	Anas hel
Betula pubescens	*Neoantrodia serialis*	*Anastrophyllum minutum*	Anas min
Calluna vulgaris	*Amyloporia sinuosa*	*Anastrophyllum michauxii*	Anas mix
Carex globularis	*Asterodon ferruginosus*	*Andreaea rupestris*	Andr rup
Carex spp.	*Byssoporia terrestris*	*Atrichum tenellum*	Atr ten
Dryopteris spp.	*Cystostereum murrayi*	*Atrichum undulatum*	Atri und
Empetrum nigrum	*Dacryobolus karstenii*	*Aulacomnium palustre*	Aula pal
Epilobium angustifolium	*Fomitopsis pinicola*	*Barbilophozia attenuata*	Barb att
Equisetum sylvaticum	*Rhodofomes roseus*	*Barbilophozia barbata*	Barb bar
Geranium sylvaticum	*Gloeophyllum sepiarium*	*Barbilophozia floerkei*	Barb flo
Goodyera repens	*Heterobasidion annosum*	*Barbilophozia hatcheri*	Barb hat
Gymnocarpium dryopteris	*Ischnoderma benzoinum*	*Barbilophozia lycopodioides*	Barb lyc
Linnaea borealis	*Steccherinum collabens*	*Blepharostoma trichophyllum*	Blep tri
Listera cordata	*Butyrea luteoalba*	*Brachythecium s.lat sp.*	Brach sp
Luzula pilosa	*Leptoporus mollis*	*Calypogeia integristipula*	Caly int
Lycopodium annotinum	*Postia caesia*	*Calypogeia muelleriana*	Caly mue
Maianthemum bifolium	*Postia fragilis*	*Calypogeia neesiana*	Caly nee
Melampyrum spp.	*Postia hibernica/parva*	*Campylophyllum sommerfeltii*	Camp som
Orthilia secunda	*Postia sericeomollis*	*Fuscocephaloziopsis affinis*	Ceph aff
Oxalis acetosella	*Postia tephroleuca/lactea*	*Cephalozia bicuspidata*	Ceph bic
Picea abies	*Pelloporus leporinus*	*Cephalozia lunulifolia*	Ceph lun
Rubus idaeus	*Phellinus chrysoloma*	*Cephalozia pleniceps*	Ceph ple
Rubus saxatilis	*Phellinus ferrugineofuscus*	*Ceratodon purpureus*	Cer pur
Solidago virgaurea	*Phellinus nigrolimitatus*	*Cirriphyllum piliferum*	Cirr pil
Sorbus aucuparia	*Fuscoporia viticola*	*Cynodontium strumiferum*	Cyno str
Trientalis europaea	*Phlebia centrifuga*	*Dicranella crispa*	Dicr cri
Vaccinium myrtillus	*Phlebiopsis gigantea*	*Dicranum drummondii*	Dicr dru
Vaccinium vitis-idaea	*Sistotrema alboluteum*	*Dicranum fuscescens s.lat*	Dicr fus
	Sistotrema muscicola	*Dicranella grevilleana*	Dicr gre
	Skeletocutis amorpha	*Dicranum majus*	Dicr maj
	Skeletocutis biguttulata	*Dicranum montanum*	Dicr mon
	Sidera lenis	*Dicranum polysetum*	Dicr pol
	Stereum sanguinolentum	*Dicranum scoparium*	Dicr sco
	Trametes ochracea	*Diplophyllum taxifolium*	Dipl tax
	Trechispora mollusca	*Distichium spp.*	Dist sp.
	Trichaptum abietinum	*Hedwigia ciliata*	Hedw cil
	Trichaptum fusco-violaceum	*Heterocladium dimorphum*	Hete dim
	Veluticeps abietina	*Hylocomium splendens*	Hylo spl
		Hylocomiastrum umbratum	Hylo umb
		Isothecium myosuroides	Isot myo

		Leiocolea heterocolpos	Leio het
**Vascular plants**	**Wood-inhabiting fungi**	**Bryophytes**	**Bryophytes abbreviation**
		Leptobryum pyriforme	Lep pyr
		Lepidozia reptans	Lep rep
		Lophozia ciliata	Loph cil
		Lophocolea heterophylla	Loph het
		Schistochilopsis incisa	Loph inc
		Lophozia longidens	Loph longid
		Lophozia guttulata	Loph longif
		Lophozia obtusa	Loph obt
		Lophozia ventricosa s.lat.	Loph ven
		Metzgeria furcata	Metz fur
		Mnium stellare	Mniu ste
		Mylia anomala	Myli ano
		Orthotrichum obtusifolium	Orth obt
		Paraleucobryum longifolium	Para lon
		Platydictya jungermannioides	Pla jun
		Plagiochila asplenioides	Plag asp
		Plagiothecium curvifolium	Plag cur
		Plagiothecium denticulatum	Plag den
		Plagiothecium laetum	Plag laet
		Plagiomnium medium	Plag med
		Plagiothecium undulatum	Plag und
		Pleurozium schreberi	Pleu sch
		Pogonatum dentatum	Pogo den
		Pogonatum urnigerum	Pogo urn
		Pohlia cruda	Pohl cru
		Pohlia nutans	Pohl nut
		Polytrichastrum alpinum	Poly alp
		Polytrichum commune s.lat.	Poly com
		Polytrichastrum formosum	Poly for
		Polytrichum juniperinum	Poly jun
		Polytrichastrum longisetum	Poly lon
		Polytrichum strictum	Poly str
		Pseudobryum cinclidioides	Pse cin
		Ptilidium ciliare	Ptil cil
		Ptilium crista-castrensis	Ptil cri
		Ptilidium pulcherrimum	Ptil pul
		Racomitrium heterostichum	Raco het
		Racomitrium microcarpon	Raco mic
		Radula complanata	Radu com
		Rhizomnium pseudopunctatum	Rhiz pse
		Rhizomnium punctatum	Rhiz pun
		Rhodobryum roseum	Rhod ros

		Rhytidiadelphus triquetrus	Rhyt tri
**Vascular plants**	**Wood-inhabiting fungi**	**Bryophytes**	**Bryophytes abbreviation**
		Riccardia latifrons	Ricc lat
		Riccardia palmata	Ricc pal
		Sanionia uncinata	Sani unc
		Scapania mucronata	Scap muc
		Scapania subalpina	Scap sub
		Sciuro-hypnum oedipodium	Sci oed
		Sciuro-hypnum reflexum	Sci ref
		Sciuro-hypnum starkei	Sci sta
		Sphagnum angustifolium	Spha ang
		Sphagnum capillifolium	Spha cap
		Sphagnum girgensohnii	Spha gir
		Sphagnum magellanicum	Spha mag
		Sphagnum palustre	Spha pal
		Sphagnum riparium	Spha rip
		Sphagnum russowii	Spha rus
		Splachnum ampullaceum	Spl amp
		Splachnum luteum	Spla lut
		Splachnum rubrum	Spla rub
		Tayloria serrata	Tay ser
		Tetraplodon angustatus	tetr ang
		Tetraplodon mnioides	Tetr mni
		Tetraphis pellucida	Tetr pel
		Tortella tortuosa	Tort tor
		Tritomaria quinquedentata	Trit qui
